# Conformational regulation of *Escherichia coli* DNA polymerase V by RecA and ATP

**DOI:** 10.1371/journal.pgen.1007956

**Published:** 2019-02-04

**Authors:** Malgorzata M. Jaszczur, Dan D. Vo, Ramunas Stanciauskas, Jeffrey G. Bertram, Adhirath Sikand, Michael M. Cox, Roger Woodgate, Chi H. Mak, Fabien Pinaud, Myron F. Goodman

**Affiliations:** 1 Department of Biological Sciences, University of Southern California, Los Angeles, California, United States of America; 2 Department of Chemistry, University of Southern California, Los Angeles, California, United States of America; 3 Department of Biochemistry, University of Wisconsin-Madison, Madison, Wisconsin, United States of America; 4 Laboratory of Genomic Integrity, National Institute of Child Health and Human Development, National Institutes of Health, Bethesda, Maryland, United States of America; 5 Center of Applied Mathematical Sciences, University of Southern California, Los Angeles, California, United States of America; 6 Department of Physics and Astronomy, University of Southern California, Los Angeles, California, United States of America; Max Planck Institute for Terrestrial Microbiology, GERMANY

## Abstract

Mutagenic translesion DNA polymerase V (UmuD′_2_C) is induced as part of the DNA damage-induced SOS response in *Escherichia coli*, and is subjected to multiple levels of regulation. The UmuC subunit is sequestered on the cell membrane (spatial regulation) and enters the cytosol after forming a UmuD′_2_C complex, ~ 45 min post-SOS induction (temporal regulation). However, DNA binding and synthesis cannot occur until pol V interacts with a RecA nucleoprotein filament (RecA*) and ATP to form a mutasome complex, pol V Mut = UmuD′_2_C-RecA-ATP. The location of RecA relative to UmuC determines whether pol V Mut is catalytically *on* or *off* (conformational regulation). Here, we present three interrelated experiments to address the biochemical basis of conformational regulation. We first investigate dynamic deactivation during DNA synthesis and static deactivation in the absence of DNA synthesis. Single-molecule (sm) TIRF-FRET microscopy is then used to explore multiple aspects of pol V Mut dynamics. Binding of ATP/ATPγS triggers a conformational switch that reorients RecA relative to UmuC to activate pol V Mut. This process is required for polymerase-DNA binding and synthesis. Both dynamic and static deactivation processes are governed by temperature and time, in which *on* → *off* switching is “rapid” at 37°C (~ 1 to 1.5 h), “slow” at 30°C (~ 3 to 4 h) and does not require ATP hydrolysis. Pol V Mut retains RecA in activated and deactivated states, but binding to primer-template (p/t) DNA occurs only when activated. Studies are performed with two forms of the polymerase, pol V Mut-RecA wt, and the constitutively induced and hypermutagenic pol V Mut-RecA E38K/ΔC17. We discuss conformational regulation of pol V Mut, determined from biochemical analysis *in vitro*, in relation to the properties of pol V Mut in RecA wild-type and SOS constitutive genetic backgrounds *in vivo*.

## Author summary

*Escherichia coli* upregulates more than 40 genes as part of the DNA damage-induced SOS regulon, many of which are involved in DNA repair and cell division. However, three DNA polymerases, pols V, II, and IV, are also induced to rescue replication forks blocked at persisting template lesions. Pol V (UmuD′_2_C), encoded by the UV mutagenesis genes (*umuDC*), is primarily responsible for the increase in UV-induced chromosomal mutagenesis. However, pol V is catalytically inert. Interaction with a RecA nucleoprotein filament (RecA*) and ATP is required to convert pol V to an activated “mutasome” complex, pol V Mut = UmuD′_2_C-RecA-ATP. Here, we show that pol V Mut deactivates dynamically during DNA synthesis, and statically in the absence of synthesis. Activated and deactivated states are governed by a conformational switch that repositions RecA relative to UmuC. Switching rates are more rapid at 37 than at 30°C, and do not require ATP hydrolysis. ATP (ATPγS) binding plays two required regulatory roles: 1) it allows binding of pol V Mut to primer-template DNA; 2) it triggers the RecA-UmuC conformational switch that activates pol V Mut.

## Introduction

DNA polymerase V (pol V) is induced as part of the SOS regulon in *Escherichia coli* in response to DNA damage [1]. Pol V is assembled as a UmuD′_2_C heterotrimeric complex. This complex is activated extremely late in the induction process, at around 45 min after exposure to either UV light or to chemicals that damage DNA [2, 3]. SOS-induced levels of pol V are about 60 molecules/cell [4]. In the absence of DNA damage, the constitutive level of pol V is barely detectable, ~ 2 molecules/cell observed by live-cell imaging [4].

Damage-induced SOS mutagenesis does not rise above spontaneous levels in the absence of pol V [5–7]. Therefore, pol V appears to be responsible for virtually all the increase in mutagenesis associated with damage-induced induction of the SOS response. This is true even though the two other SOS-induced pols II and IV are present in the cell at high constitutive levels, which increase further and rapidly (< 1 min) upon SOS induction [8, 9]. Presumably to ensure both accurate transmission of genetic information and optimal cellular viability, *E*. *coli* takes great pains to restrict pol V access to undamaged DNA through low constitutive expression. Access to damaged DNA is limited by delayed induction, and rapid proteolysis of the Umu proteins [10], thus affording ample time for the error-free repair of DNA templates to occur prior to calling upon error-prone pol V-catalyzed translesion synthesis (TLS).

The temporal control of pol V is just one facet of a highly complex scheme, which encompasses three additional regulatory processes, spatial [4], conformational [11, 12], and internal [13]. Spatial regulation was recently revealed by live-cell imaging studies and entails the synthesis and sequestering of the UmuC subunit on the cell membrane [4]. Release of pol V into the cytosol requires binding to UmuD′_2_ [4]. However, pol V in the form of UmuD′_2_C is catalytically “dead” [11, 13–15]. A subsequent 2-step activation process involving transfer of a RecA monomer from the 3′-proximal tip of a RecA nucleoprotein filament (RecA*) to form UmuD′_2_C-RecA, and then binding a molecule of ATP, is required to produce a catalytically active pol V “mutasome”, pol V Mut = UmuD′_2_C-RecA-ATP [11, 13]. Conformational regulation entails serial conversions of a pol V Mut complex from (i) an initially catalytically inactive state that is unable to bind to primer/template (p/t) DNA to (ii) an activated state that copies DNA to (iii) a deactivated state that halts further synthesis. The RecA subunit of pol V Mut is retained in both activated and deactivated states [11]. Conformational regulation appears to be governed by reorientation of RecA relative to UmuC and UmuD′_2_ [12]. Internal regulation, on the other hand, is marked by the presence of a bound ATP and a DNA dependent ATPase activity unique to pol V Mut [13]. The intrinsic pol V Mut ATPase is distinct from the canonical DNA-dependent ATPase of RecA* [13].

The presence of a stably bound ATP, or slowly hydrolysable ATPγS molecule, is necessary for binding pol V Mut to primer-template (p/t) DNA [13]. The RecA subunit modulates conformational and internal regulation through its contact points with UmuC and UmuD′_2_, and its interaction with ATP [12]. Pol V Mut can be assembled with wild-type RecA and with RecA mutants that exhibit widely different SOS mutagenic phenotypes, thus providing a unique opportunity to relate biochemical to genetic data. In 1982, Witkin and colleagues identified strains of *E. coli* with mutations in *recA* that induced SOS constitutively and caused a ~100-fold increase in spontaneous SOS mutagenesis [16]. One particularly active allele is *recA730* [17], which was subsequently shown to harbor an E38K substitution (RecA E38K) [18].

Here, we explore conformational regulation mechanisms revealing how RecA and ATP function in the mutasome as a temperature-dependent *on-off* toggle switch. The experiments are performed using combinations of pol V Mut assembled with wild-type RecA (pol V Mut wt) and with an SOS constitutive RecA mutant containing an E38K amino acid substitution and a deletion of 17 amino acids at the C terminus (pol V Mut E38K/ΔC17).

## Results

Pol V Mut = UmuD′_2_C-RecA-ATP exists in either activated or deactivated conformational states. Pol V Mut can be assembled in an active conformational state by incubating UmuD′_2_C with RecA* and ATP or ATPγS [11, 13]. Pol V Mut deactivates in either of two ways, “dynamically” during DNA synthesis or “statically” in the absence of DNA synthesis [11]. Neither pol V (UmuD′_2_C) nor pol V Mut (UmuD′_2_C-RecA) lacking a bound ATP, are able to synthesize DNA; binding to p/t DNA requires a stably bound nucleotide cofactor (ATP/ATPγS) as an integral component of the mutasome complex [13]. Here, we address two essential properties of pol V Mut regulation: 1) the distinctive biochemical properties of dynamic and static polymerase deactivation; 2) the roles of ATP, ATPγS, and RecA in pol V Mut-DNA binding, observed at single-molecule resolution using TIRF-FRET microscopy.

### Pol V Mut dynamic deactivation

**Fig 1:**
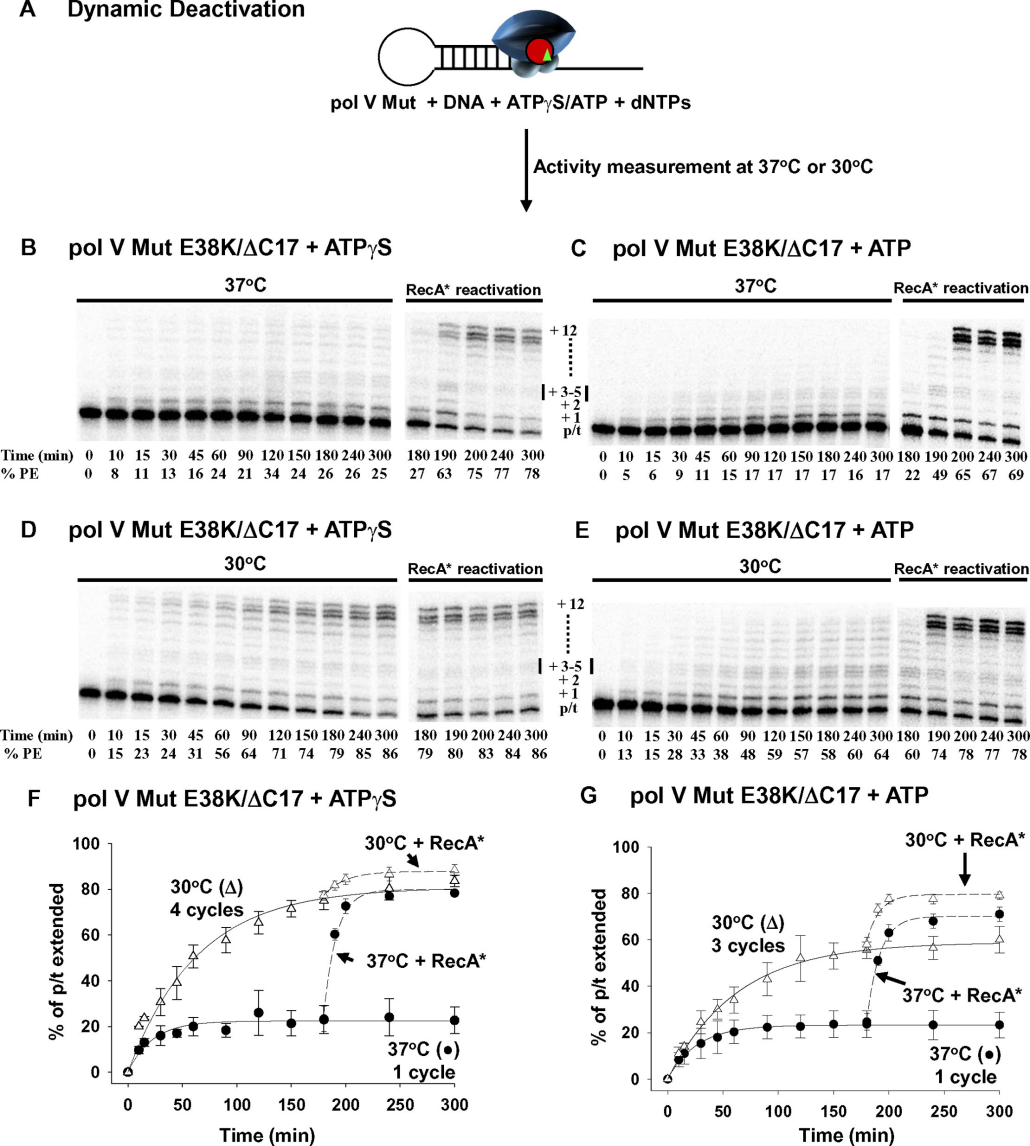
Temperature-dependent dynamic deactivation of pol V Mut E38K/ΔC17. (A) Sketch showing dynamic deactivation of pol V Mut E38K/ΔC17 (200 nM) with ATPγS /ATP (500 μM) at 37°C and 30°C in the presence of saturating concentration of dNTP’s (mix of dTTP, dCTP, dGTP 500 μM each). Representative DNA synthesis gels for pol V Mut E38K/ΔC17 with ATP/ATPγS at 37°C and 30°C are presented in (B-E). Each experiment was repeated 3 times, and the average % PE (percent p/t DNA extended) with the SD (standard deviation) for each reaction time point is graphed in panels (F-G). Pol V Mut E38K/ΔC17 deactivates in about 1.5 h at 37°C and completes only one round of DNA synthesis with ATP/ATPγS (B-C and F-G black circles in the graphs). In contrast, at 30°C, pol V Mut E38K/ΔC17 deactivates in about 3h, which allows the enzyme to complete 4 rounds of DNA synthesis with ATPγS and 3 rounds with ATP (D-E and F-G white triangles in the graphs). Deactivated pol V Mut E38K/ΔC17 is not “dead” and is reactivated by adding RecA* (200 nM; RecA* in B-G). % PE refers to percent p/t DNA extended and was calculated as the integrated gel band intensities of extended hairpin DNA over total DNA intensity.

DNA polymerases normally act in accord with sequential bisubstrate kinetics by binding first to p/t DNA and then to dNTP substrates. One, or multiple dNMP incorporations can occur during a single DNA binding event, which is followed by dissociation with the subsequent re-initiation of synthesis on another DNA molecule. Continuing pol-DNA cycling occurs until most of the DNA has been copied. However, unlike generic pols, pol V Mut behaves differently, depending on reaction temperature. Pol V Mut carries out just a single-round of DNA synthesis at 37°C, whereas multiple cycles occur at 30°C (Fig 1). The more active pol V Mut E38K/ΔC17 was exploited for many of these experiments as indicated.

To measure the number of cycles of DNA synthesis, we incubated pol V Mut E38K/ΔC17 with either ATPγS (Fig 1B, 1D and 1F), or ATP (Fig 1C, 1E and 1G), using a 5-fold molar excess of p/t DNA over polymerase. Pol V Mut E38K/ΔC17 is limited to just one round of replication (20% p/t DNA elongation) with either ATPγS or ATP at 37°C (Fig 1F and 1G). Although pol V E38K/ΔC17 Mut is deactivated following one round of synthesis at 37°C, the enzyme is clearly not dead but is instead temporarily inactive, since substantial reactivation of the polymerase occurs, with ~ 75 to 85% p/t DNA subsequently extended following exposure to RecA* (Fig 1F and 1G). In contrast, pol V Mut E38K/ΔC17 catalyzes multiple rounds of synthesis at 30°C (Fig 1F and 1G). Pol V Mut E38K/ΔC17 performs four rounds of DNA synthesis at 30°C with ATPγS (Fig 1F), with the extent of polymerase cycling remaining at 4 when the ratio of p/t DNA to polymerase is increased from 5-fold to 10-fold molar excess (S1 Fig). When ATP is used instead of ATPγS, Pol V Mut E38K/ΔC17 performs 3 rather than 4 rounds of DNA synthesis at 30°C (Fig 1G).

The dynamic deactivation of pol V Mut is rapidly reversed by the addition of *trans*RecA* at 37 and 30°C with either ATPγS or ATP. Reactivation is caused by a continual replenishment of activated pol V Mut [11, 19] irrespective of the extent of cycling. There is a clear distinction to be made between polymerase deactivation and inactivation. Pol V Mut E38K/ΔC17 and pol V Mut wt are inactivated following incubation at 45°C for 15 min and cannot be reactivated by the addition of RecA* (S2B and S2E Fig). In contrast, pol V (S2C Fig) and RecA (S2D and S2F Fig) when incubated alone at 45°C, remain functionally active since they retain the ability to assemble into an activated form of pol V Mut.

Synthesis with ATPγS appears to be processive at 30°C, with most of the elongation gel bands extending either to the end of the template (12 nt) or terminating one base prior to the end (Fig 1D). Synthesis with ATPγS at 37°C is more limited (Fig 1B), with only about 20% of the primers being extended under conditions where the p/t DNA substrate is in 5-fold excess to the enzyme. Thus, only one synthetic cycle is occurring. Product lengths vary, and the observed processive deoxynucleotide additions are taking place on the same p/t DNA molecule. For the same reason, i.e., absence of cycling, processive synthesis appears to be occurring with ATP at 37°C, even though extension is limited to < 5 nt (Fig 1C). However, with ATP at 30°C synthesis is essentially distributive as shown by the presence of a decreasing gradient of small to large primer elongation bands, along with far fewer primers that are extended to the end of the template (Fig 1E).

**Fig 2:**
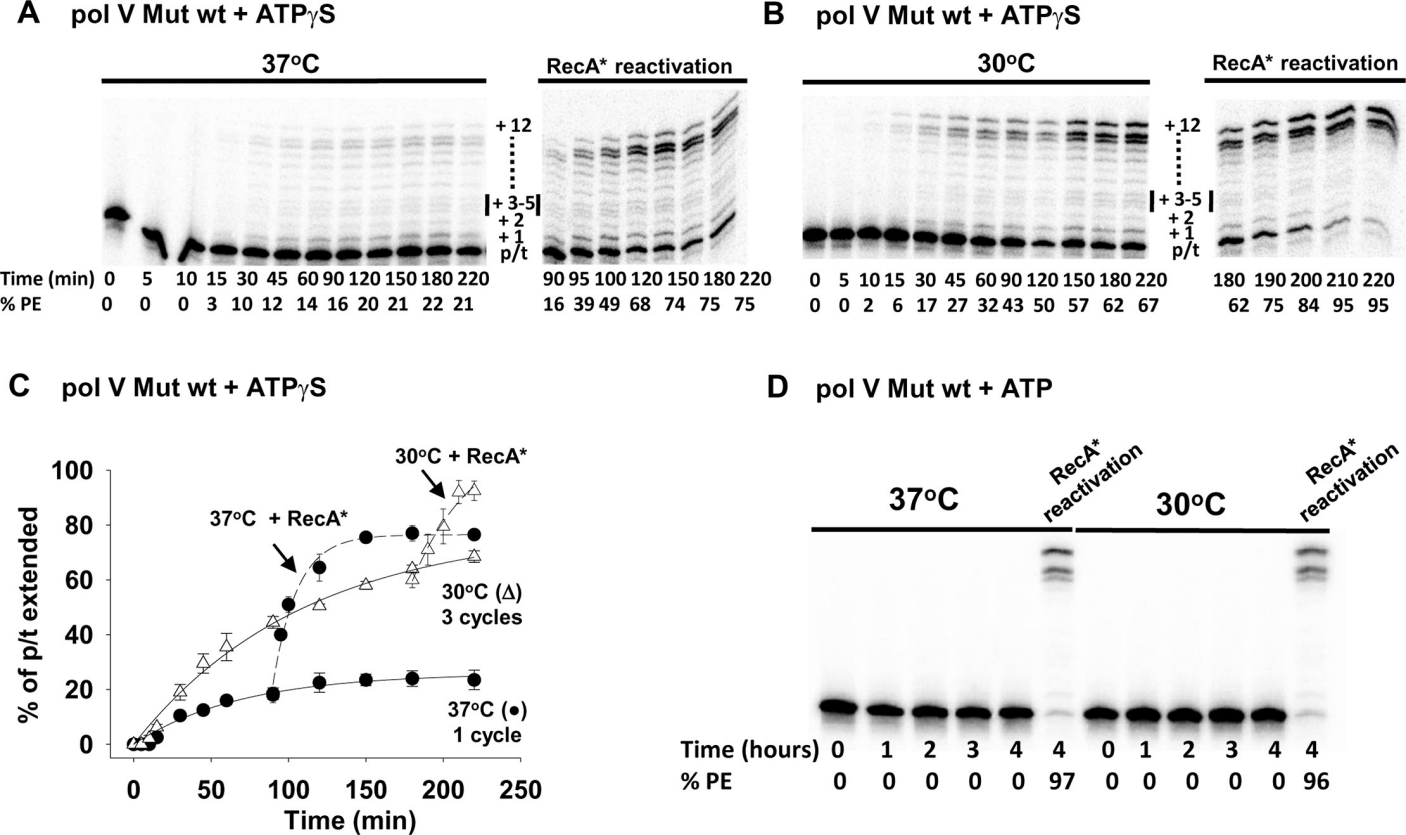
Temperature-dependent dynamic deactivation of pol V Mut wt. Activity and dynamic deactivation of pol V Mut wt (200 nM) were measured as shown in Fig 1A at (A) 37°C and (B) 30°C on 12 nt oh HP (1 μM) in the presence of saturating concentration of ATPγS (500 μM) and dNTP’s (mix of dTTP, dCTP, dGTP 500 μM each). (C) Prior to deactivation, pol V Mut wt performs one round of DNA synthesis at 37°C (black circles in the graph) and 3 rounds at 30°C (white triangles in the graph). Deactivated pol V Mut is reactivated by adding RecA* wt (200 nM). (D) Activity of pol V Mut wt measured in the presence of ATP (500 μM) and dNTP’s (dTTP, dCTP, dGTP 500 μM each). Pol V Mut wt is not active with ATP but it can synthesize DNA when *trans*-activated by RecA* wt (*trans*-activation reaction was performed for 30 min). Representative DNA synthesis gels for pol V Mut wt are presented in (A, B, and D). The experiments were repeated 3 times and the average % PE (percent p/t DNA extended) with the SD (standard deviation) for each reaction time point is graphed in panel C.

**Fig 3:**
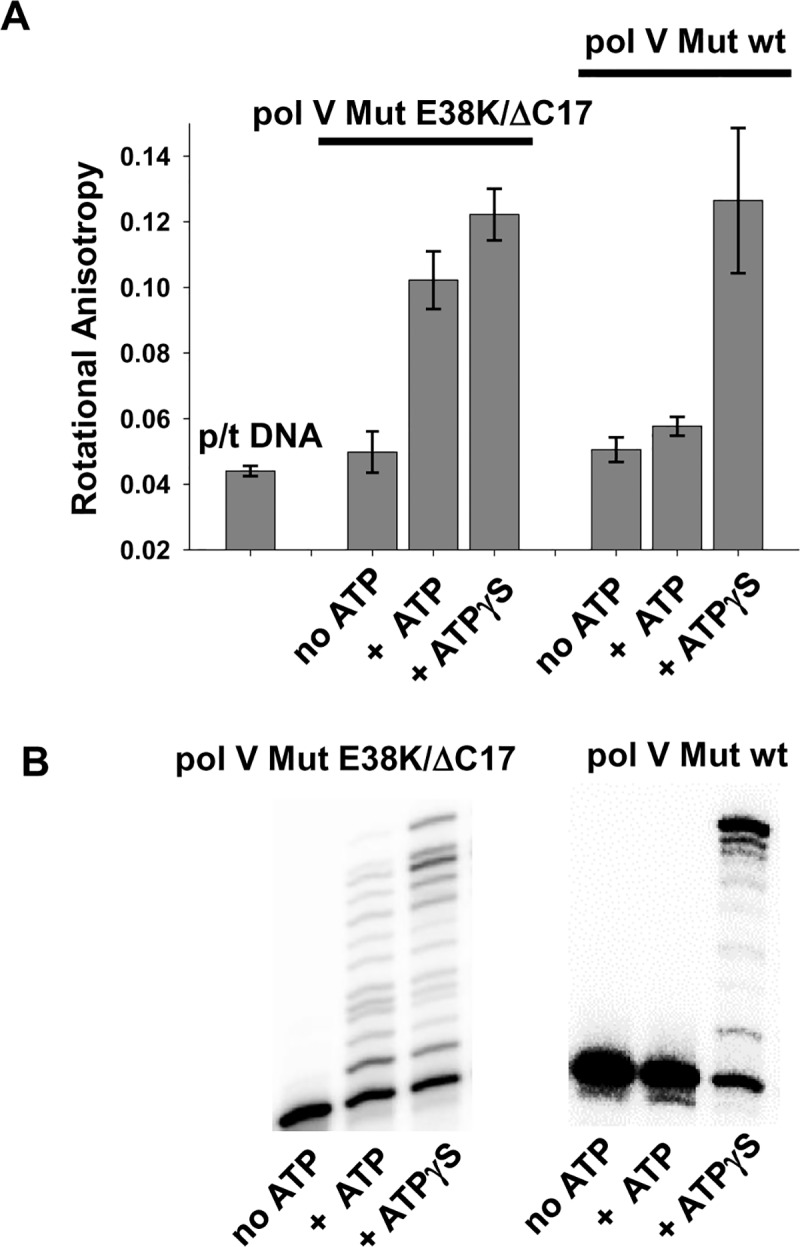
ATP is required for Pol V Mut p/t DNA binding and activity. (A) Binding of pol V Mut (400 nM) to 12 nt oh HP DNA (50 nM) is measured as a change in rotational anisotropy in the presence and absence of saturating concentration of ATP/ATPγS (500 μM). The graph presents the average and SD for four independent rotational anisotropy measurements (B) Activity of pol V Mut E38K/ΔC17 (200 nM) and pol V Mut wt (200 nM) is measured on 12 nt oh HP (50 nM) in the absence and presence of ATP/ATPγS (500 μM) and dNTP’s (mix of dTTP, dCTP, dGTP 500 μM each). Pol V Mut can bind and synthesize DNA only in the presence of ATP/ATPγS.

Pol V Mut wt performs three rounds of DNA synthesis at 30°C (Figs 2B and 1C) but is restricted to a single round at 37°C (Fig 2A and 2C), as observed for pol V Mut E38K/ΔC17 (Fig 1F). In a similar manner, synthesis appears to be processive in the presence of ATPγS, and RecA* reactivation occurs at both temperatures (Fig 2A and 2B). However, a definitive difference in properties of the two forms of pol V Mut is that pol V Mut wt cannot synthesize DNA in the presence of ATP (Fig 2D) *in vitro*. Concomitantly, binding to p/t DNA is weak in the presence of ATP (Fig 3A, ~20% increase in rotational anisotropy). Pol V Mut wt activity is robust with ATPγS (Fig 2A and 2B), corresponding to a much stronger p/t DNA binding (Fig 3A, ~2.5-fold increase in rotational anisotropy). Pol V Mut E38K/ΔC17, binds much more strongly to DNA with either ATP or ATPγS (Fig 3A, ~2.8-fold and ~3-fold increase in rotational anisotropy, respectively), and performs robust DNA synthesis (Fig 1). Neither form of pol V Mut binds to DNA in the absence of ATP/ATPγS (Fig 3A), thus precluding DNA synthesis (Fig 3B).

The halt in DNA synthesis after a prescribed number of cycles of DNA synthesis defines dynamic deactivation. The dynamic deactivation profiles (Fig 1F and 1G and Fig 2C) can be analyzed using two parameters, a pol V Mut intrinsic DNA synthesis rate constant (*k*), and a deactivation rate (*D*) (S3 Fig). The synthesis rate constants for pol V Mut E38K/ΔC17 are *k* ~ 0.008 min^-1^ for ATPγS and ATP at 37°C, and about 1.5-fold faster at 30°C. Pol V Mut wt with ATPγS, behaves in a similar manner, *k* ~ 0.004 min^-1^ at 37°C and about 1.5-fold faster at 30°C. Using pol V Mut E38K/ΔC17-ATPγS, we also determined whether or not the presence of the β-sliding processivity clamp altered the dynamic deactivation profile, and found that it had no measurable effect (S4 Fig).

The deactivation rate (*D*) is the key parameter that characterizes the conformational regulation mechanism. At 37°C, the value of *D* is similar for pol V Mut E38K/ΔC17 (*D* = 0.028 min^-1^), pol V Mut wt (*D* = 0.015 min^-1^) with ATPγS (S3A and S3E Fig), and also for pol V Mut E38K/ΔC17 with ATP (*D* = 0.026 min^-1^) (S2C Fig). At 30°C, the deactivation rate constant (*D*) is reduced by about 3 to 5-fold compared to 37°C for pol V Mut E38K/ΔC17 (*D* = 0.01 min^-1^) with ATPγS and ATP (S3B and S3D Fig), and pol V Mut wt (*D* = 0.003) with ATPγS (S3F Fig).

### Pol V Mut static deactivation

**Fig 4:**
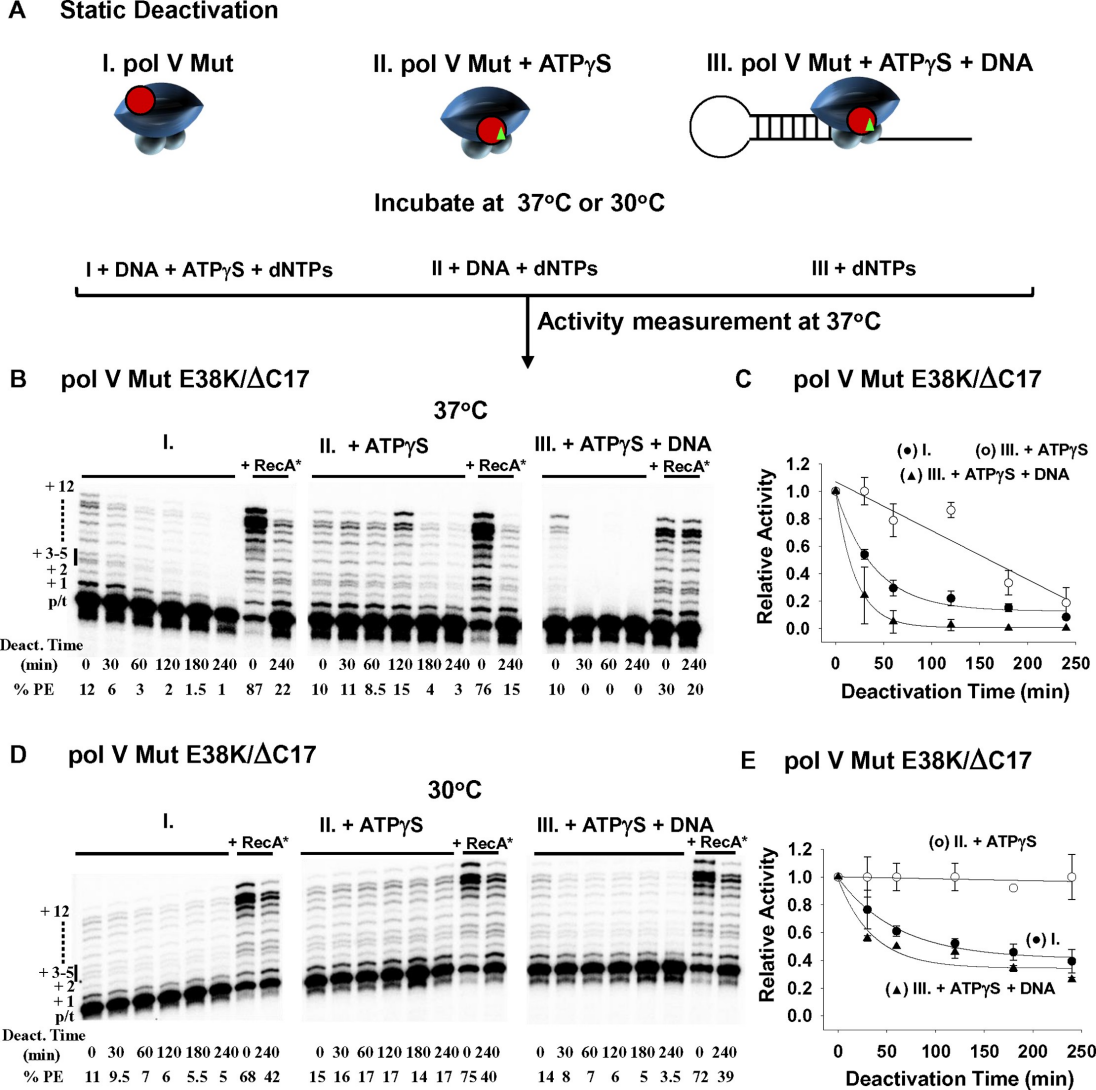
Static Deactivation of pol V Mut E38K/ΔC17 at 37°C and 30°C. (A) Sketch showing static deactivation of pol V Mut E38K/ΔC17 (200 nM) in the absence of DNA synthesis at 37°C and 30°C. Static deactivation was determined by measuring the extent of DNA synthesis as a function of incubation time on 12 nt oh HP p/t DNA at 37°C in the presence of ATPγS and dNTP’s (dTTP, dCTP, dGTP 500 μM each). Pol V Mut E38K/ΔC17 was incubated either alone (I.) or with ATPγS (II.), or with ATPγS and 12nt oh HP DNA (III.). (B) and (D) show representative gels for each deactivation condition. Pol V Mut E38K/ΔC17 deactivates more slowly at 30°C (D-E) compared to 37°C (B-C). At each temperature, pol V Mut is stabilized when incubated in the presence of ATPγS. Deactivated pol V Mut E38K/ΔC17 is not dead since it can be reactivated by incubation with RecA* (+RecA*). Static deactivation is expressed as the relative polymerase activity measured at each incubation time point divided by the polymerase activity measured at t = 0. Each experiment was repeated 2–3 times and average relative activity along with SD for each deactivation time point are presented in (C) and (D). (I. and black circles in the graphs) represent static deactivation of pol V Mut alone, (II. and white circles in the graphs) represent deactivation of pol V Mut +ATPγS and (III. and black triangles in the graphs) represent deactivation of pol V Mut + ATPγS + 12 nt oh HP.

When assembled in an activated state, pol V Mut E38K/ΔC17 undergoes rapid dynamic deactivation during DNA synthesis at 37 and 30°C in the presence of either ATPγS or ATP (Fig 1). Deactivation of Pol V Mut in the absence of DNA synthesis is defined as static deactivation. Pol V Mut E38K/ΔC17 was incubated at either 37 or 30°C for varying lengths of time either alone, or + ATPγS, or ATPγS + DNA (Fig 4A). Deoxynucleoside triphosphates were omitted in these incubations, precluding DNA synthesis. At each time point, remaining enzyme activity was assessed at 37°C by the addition of any components needed for DNA synthesis, ATPγS, p/t DNA, and dNTP substrates (Fig 4A). Pol V Mut E38K/ΔC17 deactivates rapidly (0.015 min^-1^) at 37°C (S5A Fig), losing ~ 80% of its activity in about 1 h, while retaining a residual level of activity at 4 h (Fig 4B and 4C). In contrast, pol V Mut E38K/ΔC17 is considerably more stable at 30°C, losing less than half of its activity (0.008 min^-1^) (S5A Fig), with about 60% activity retained after a 1 h incubation, decreasing to ~ 50% after 4 h (Fig 4D and 4E). Pol V Mut E38K/ΔC17 is partially stabilized in its activated state at 37°C in the presence of ATPγS, whereas deactivation occurs more rapidly in the presence of ATPγS + p/t DNA (Fig 4B and 4C). At 30°C, pol V Mut is fully stabilized by ATPγS, while a moderate rate of deactivation occurs with ATPγS + p/t DNA (Fig 4D and 4E).

**Fig 5:**
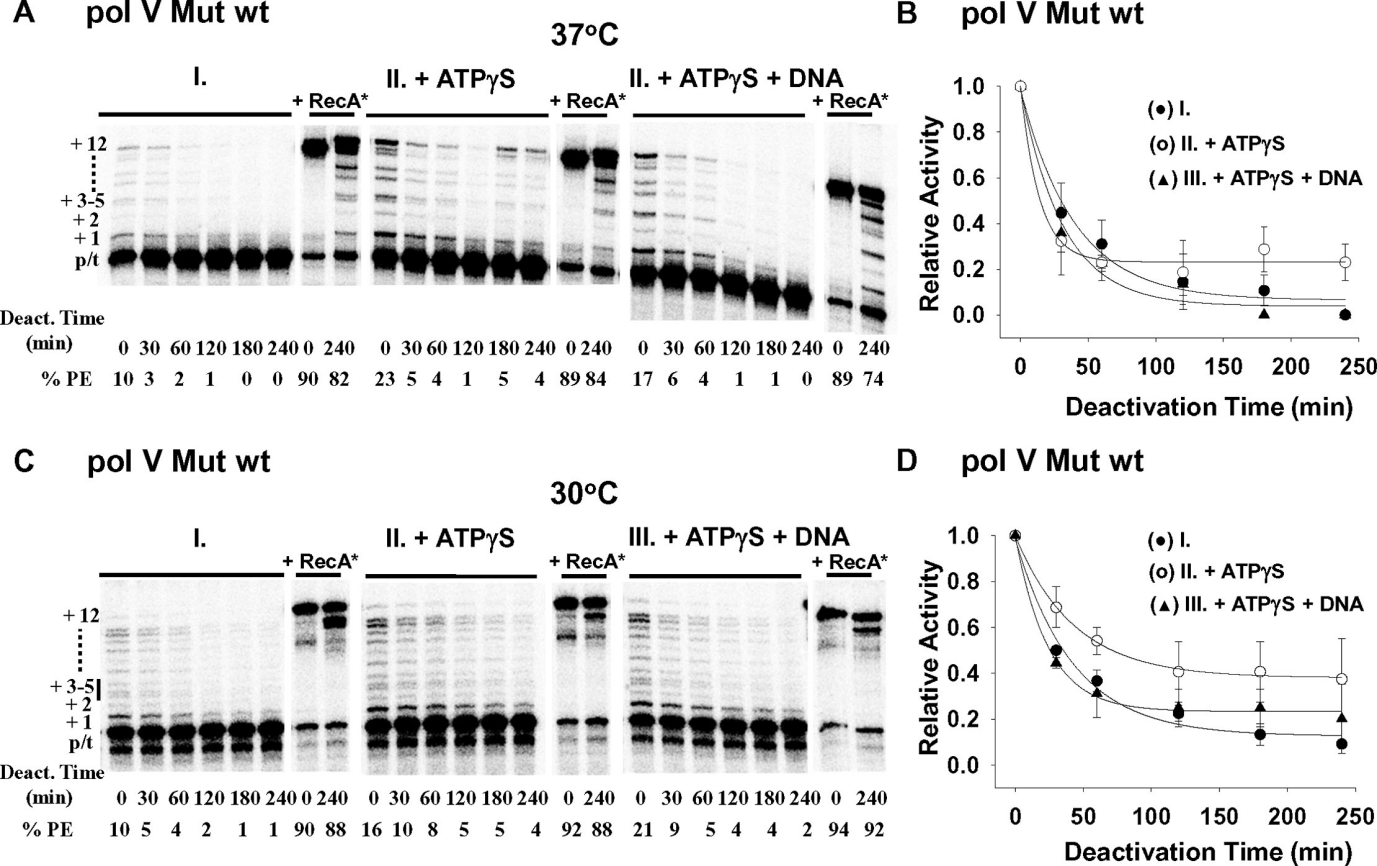
Static deactivation of pol V Mut wt at 37°C and 30°C. Static deactivation of pol V Mut wt was measured as shown in Fig 4A. Pol V Mut wt deactivates statically in the absence of DNA synthesis at 37°C (A-B) and 30°C (C-D). Pol V Mut wt was incubated either alone (I. and black circles in the graphs) or with ATPγS (II. and white circles in the graphs), or with ATPγS and 12nt oh HP DNA (III. and black triangles in the graphs). The static deactivation conditions and analysis are described in the legend for Fig 4. Pol V Mut wt deactivates more slowly at 30°C (C-D) compared to 37°C (A-B). However, in contrast to pol V Mut E38K/ΔC17 (Fig 4), the degree of stabilization with ATPγS is much less for pol V Mut wt. Deactivated pol V Mut wt is reactivated by incubation with RecA* (+RecA*).

Similarities and differences are observed in static deactivation rates in the presence of ATP ± p/t DNA (S6A and S6B Fig) compared to ATPγS ± p/t DNA (Fig 4). Stabilization of pol V Mut E38K/ΔC17 occurs with ATP, with greater stabilization at 30 vs 37°C (S6A vs S6B Fig), which is qualitatively similar to ATPγS (Fig 4). However, the degree of stabilization is far weaker with ATP and the addition of DNA further destabilizes the ATPγS/ATP-stabilized deactivation rates at both temperatures (Fig 4). As observed for dynamic deactivation (Fig 1), substantial RecA* reactivation of pol V Mut E38K/ΔC17 occurs for all static deactivation conditions, pol V Mut E38K/ΔC17, pol V Mut E38K/ΔC17 ± ATPγS/ATP ± p/t DNA (Fig 4, RecA*). The static deactivation properties of pol V Mut wt (Fig 5) are similar to pol V Mut E38K/ΔC17 (Fig 4). Pol V Mut wt deactivates somewhat more slowly (0.017 min^-1^) at 30°C compared to 37°C (0.021 min^-1^) (Fig 5 and S5B Fig). In contrast to pol V Mut E38K/ΔC17 (Fig 4), ATP/ATPγS weakly stabilizes pol V Mut wt (Fig 5, S6C Fig). Perhaps pol V Mut wt has more conformational flexibility than pol V Mut E38K/ΔC17, thus allowing more rapid deactivation, and more efficient reactivation.

**Fig 6:**
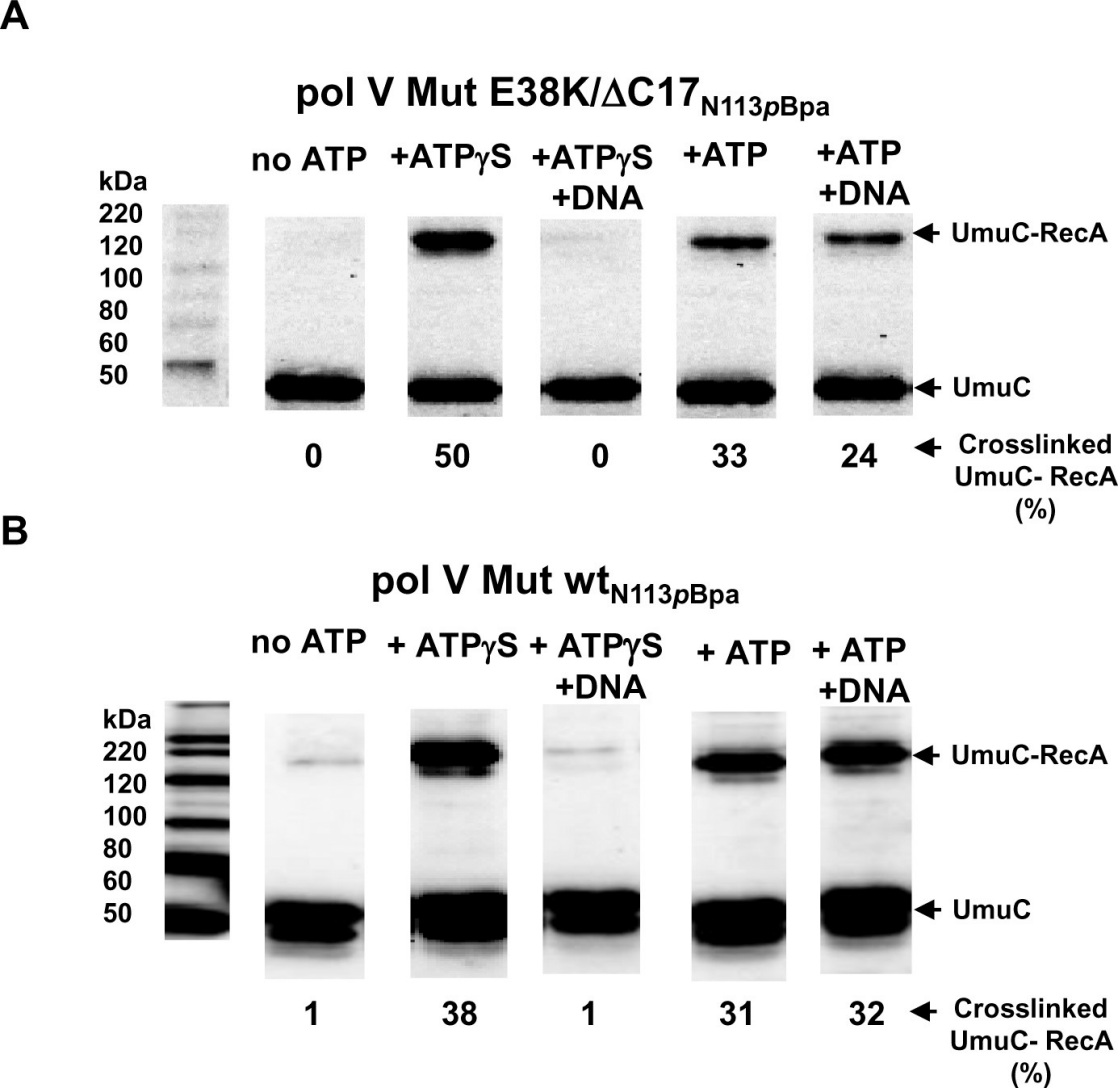
ATP and DNA induced conformational changes of pol V Mut. Crosslinking and western blot analysis using anti-UmuC antibody demonstrate conformational changes within pol V Mut E38K/ΔC17 (A) and pol V Mut wt (B) that result from binding first to ATPγS/ATP (500 μM), and then to p/t DNA DNA (5 μM) in the presence of ATPγS/ATP. Pol V Mut was assembled with crosslinkable (A) RecA E38K/ΔC17_N113*p*Bpa_ and (B) RecA wt_N113*p*Bpa_. Crosslinking experiments were repeated 4 times and the % of the UmuC-RecA crosslinking is shown below each Western blot and in S7 Fig.

To determine if ATPγS/ATP triggers conformational switching within pol V Mut, we measured UmuC-RecA crosslinking for each static deactivation condition (see methods). We assembled pol V Mut with a crosslinkable variant of RecA wt or RecA E38K/ΔC17, containing a *p*-benzoyl-l-phenylalanine (*p*Bpa) residue at aa 113, and performed UV crosslinking ± ATPγS/ATP ± p/t DNA. Two conformational changes were observed, the first upon addition of ATPγS or ATP, the second upon addition of ATPγS and p/t DNA (Fig 6).

The UmuC subunit of pol V Mut E38K/ΔC17 shows no crosslinking with RecA E38K/ΔC17 (Fig 6A and S7A Fig), and weak crosslinking with RecA wt of pol V Mut wt (Fig 6B and S7B Fig). In contrast, strong UmuC-RecA crosslinking bands are observed when ATPγS or ATP was bound to either form of pol V Mut, which places N113 residue of RecA in close proximity with UmuC (Fig 6, S7 Fig). Therefore, binding of ATPγS/ATP induces a conformational change that reorients RecA relative to UmuC.

After binding to ATPγS, binding to p/t DNA induces a second conformational change that eliminates UmuC-RecA crosslinking for both pol V Mut E38K/ΔC17 or pol V Mut wt (Fig 6, S7 Fig). When ATP is used, this second conformational change triggered by p/t DNA is not evident for pol V Mut wt, at least as measured by UmuC-RecA crosslinking (Fig 6B). This result corresponds to the weak binding of pol V Mut wt to p/t DNA in the presence of ATP measured by the small increase in rotational anisotropy (from about 0.045 to 0.05, Fig 3A). With ATP and pol V Mut E38K/ΔC17, the second conformation change is represented by a weak, yet detectable reduction in UmuC-RecA crosslinking upon p/t DNA addition (Fig 6A and S7A Fig). The range of reduction in crosslinking of pol V Mut E38K/ΔC17 in the presence of ATP+DNA compared to ATP alone is 6–11% (Fig 6A and S7A Fig), which was determined by repeating this experiment four times. Although, we would have expected to observe a stronger reduction in crosslinking, since ATP supports pol V Mut E38K/ΔC17 p/t DNA binding (Fig 3A) and DNA synthesis (Fig 1B and Fig 3B), the binding and synthesis are moderately stronger in the presence of ATPγS (Fig 3). Pol V Mut E38K/ΔC17 and pol V Mut wt are not active with ADP [13], AMP or AMPPNP (S8A and S8C Fig). The addition of ADP to either form of pol V Mut induces UmuC-RecA crosslinking. However, since pol V Mut E38K/ΔC17 and pol V Mut wt are not active with ADP there is no change in crosslinking when p/t DNA is also added (S8B and S8D Fig). No UmuC-RecA crosslinking is observed when AMP and AMPPNP is added to either form of pol V Mut (S8B and S8D Fig). We have introduced another crosslinkable residue in pol V Mut at position F21 of RecA E38K/ΔC17 (S9A and S9B Fig) and RecA wt (S9C and S9D Fig) that forms a covalent linkage with UmuD′ (S9A and S9C Fig), but not with UmuC (S9B and S9D Fig). In this case, there are no discernible conformational shifts, since the antibody band intensities remain the same ±ATPγS, and in the presence of DNA (S9A and S9C Fig). In contrast, the N113 residue of RecA does not crosslink with UmuD′ (S8E Fig) [12].

### Roles of ATP/ATPγS and RecA in pol V Mut-DNA binding observed with single-molecule FRET microscopy in real-time

**Fig 7:**
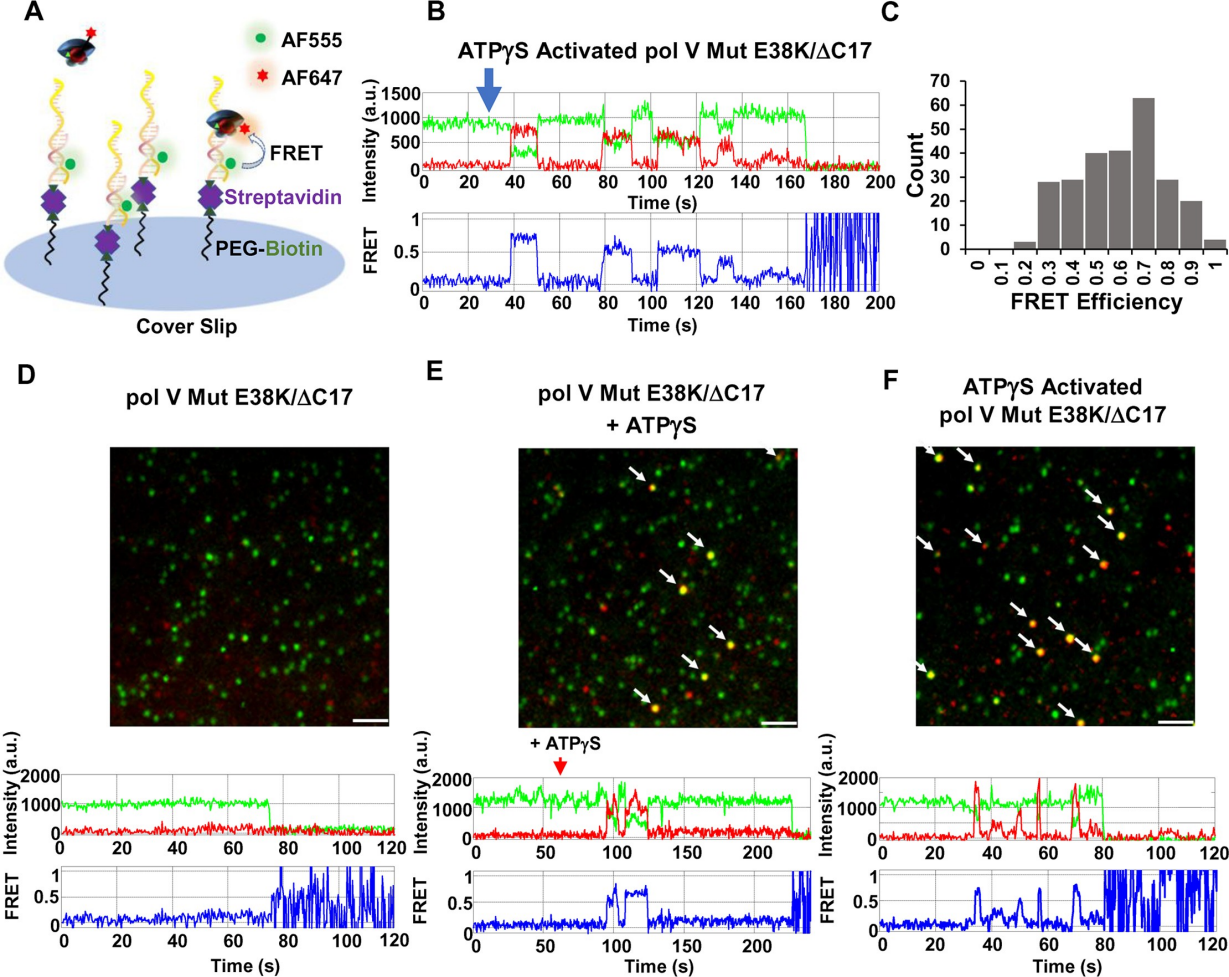
Pol V Mut binding to p/t DNA visualized at single-molecule resolution in real-time. (A) Sketch of smFRET experimental setup. An AF555 donor-labeled p/t DNA linked to streptavidin-biotin is attached to a glass slide surface. AF647 acceptor-labeled pol V Mut is then added, and DNA binding is observed as an increase in acceptor fluorophore emission that counter-correlates with a drop of a donor emission. (B) A representative smFRET trajectory showing multiple binding and unbinding events of ATPγS-activated pol V Mut E38K/ΔC17 (green = donor, red = acceptor, blue = FRET efficiency). ATPγS-activated pol V Mut was added at t = 30 s after the start of image acquisition. Data were collected for up to 3 min, prior to the onset of photobleaching. (C) Histogram representing smFRET efficiencies corresponding to the binding of ATPγS-activated pol V Mut E38K/ΔC17 to AF555-labeled p/t DNA. FRET efficiency is calculated as E = I_A_ / (I_D_+I_A_), where I_A_ and I_D_ represent acceptor and donor emission respectively. (D-F) Representative smFRET images are shown along with representative individual FRET trajectories of ATPγS-dependent binding of pol V Mut E38K/ΔC17 to p/t DNA. AF555-labeled p/t DNA is shown as green spots, and unbound AF647-labeled pol V Mut E38K/ΔC17 is shown as red spots. The pol V Mut E38K/ΔC17-p/t DNA binding events are shown as colocalized pol V Mut E38K/ΔC17 and p/t DNA signals (yellow/orange spots). Pol V Mut E38K/ΔC17 (D-E) or ATPγS activated pol V Mut E38K/ΔC17 (F) is added at t = 30 s after the start of image acquisition, followed by addition of ATPγS (t = 60 s, middle panel). Pol V Mut does not bind p/t DNA in the absence of ATPγS (D and S1 Movie). The addition of ATPγS activates pol V Mut E38K/ΔC17, resulting in binding to p/t DNA (E and S2 Movie) and pol V Mut E38K/ΔC17-p/t DNA binding events are indicated by the arrows. If pol V Mut is activated by ATPγS prior to addition to p/t DNA (F and S3 Movie), multiple and rapid p/t DNA binding events occur, indicated by arrows. The images shown in (D-F) are smFRET data integrated over 1 min following pol V Mut addition (left panel) or first binding events (middle and right panels). Scale bar is 150 mm.

Rotational anisotropy measurements using fluorescently labeled p/t DNA have established that ATP or ATPγS must be present in a complex with pol V Mut to allow the polymerase to bind to DNA (Fig 3) [13]. To investigate the molecular basis for the substantial differences in the effects of ATP/ATPγS, and wt and mutant RecA’s, on pol V Mut binding and catalysis (Fig 1, Fig 2 and Fig 3), we measured time-resolved polymerase-p/t DNA binding rates and lifetimes. We have used smFRET to visualize the dynamics of pol V Mut ± ATP/ATPγS, diffusing free in aqueous solution and subsequently binding to and releasing from DNA. An Alexa Fluor 555 (AF555) fluorescent donor, located internally on the DNA template strand, is annealed to a primer tethered to a glass coverslip, and an Alexa Fluor 647 (AF647) fluorescent acceptor is placed on the RecA subunit of pol V Mut (Fig 7A, sketch; see Methods). Donor-acceptor counter-correlated fluorescent signals (Fig 7B; upper panel) are detected when pol V Mut binds to DNA and are used to calculate FRET efficiencies (Fig 7B, lower panel and Fig 7C, histogram). The distribution of FRET efficiencies (0.2–1.0, peak at 0.7, Fig 7C) corresponds to a range of binding distances of 49 to 64 Å between AF555 on p/t DNA and AF647 on the RecA subunit of pol V Mut E38K/ΔC17; 51 Å is the R_o_ value of the FRET pair. This relatively broad distribution may reflect internal fluctuations in the location of RecA within the mutasome.

To visualize the dynamics of pol V Mut-p/t DNA binding and release, three incubations were performed for pol V Mut containing either RecA E38K/ΔC17 or RecA wt subunits: Incubation 1) pol V Mut (UmuD′_2_C-RecA) added at t = 30 s; Incubation 2) pol V Mut (UmuD′_2_C-RecA) added at t = 30 s followed by addition of ATP or ATPγS at t = 60 s; Incubation 3) activated pol V Mut (UmuD′_2_C-RecA-ATP/ATPγS) added at t = 30 s.

For incubation 1, pol V Mut E38K/ΔC17 shows no detectable binding events in the absence of nucleotide cofactors, for the entire 3 min duration of data acquisition, a time period after which onset of pol V Mut photobleaching is observed (Fig 7D). The locations of pol V Mut E38K/ΔC17 on the coverslip surface (red dots) do not colocalize with tethered p/t DNA (Fig 7D and S1 Movie).

For incubation 2 with pol V Mut E38K/ΔC17, no pol V Mut binding is initially detected between t = 30s and t = 60s (S2 Movie), as in incubation 1. However, clear binding events, characterized by the colocalization of pol V Mut with p/t DNA and by smFRET signals, are observed 30 seconds after the addition of ATPγS at t = 60 s (yellow dots, Fig 7E and S2 Movie). These data demonstrate that pol V Mut E38K/ΔC17 binds to p/t DNA only after binding ATP or ATPγS. The 30 s delay time for ATPγS pol V Mut E38K/ΔC17 binding to p/t DNA is attributable to a combination of the diffusion time required for ATPγS to bind to and activate pol V Mut E38K/ΔC17, and for activated pol V Mut E38K/ΔC17 to then bind to p/t DNA.

For incubation 3, we preassembled activated pol V Mut E38K/ΔC17 containing bound ATPγS, in 1:1 stoichiometry, and detect numerous binding events (yellow dots, Fig 7F and S3 Movie) determined by the rapid appearance of smFRET signals at t = 5 s after enzyme addition at t = 30s (Fig 7B, Fig 7F and S3 Movie). Therefore, a necessary and sufficient condition for mutasome binding to p/t DNA is that ATP/ATPγS must be present as part of an intact UmuD′_2_C-RecA-ATP/ATPγS complex in an *activated* conformational state.

**Fig 8:**
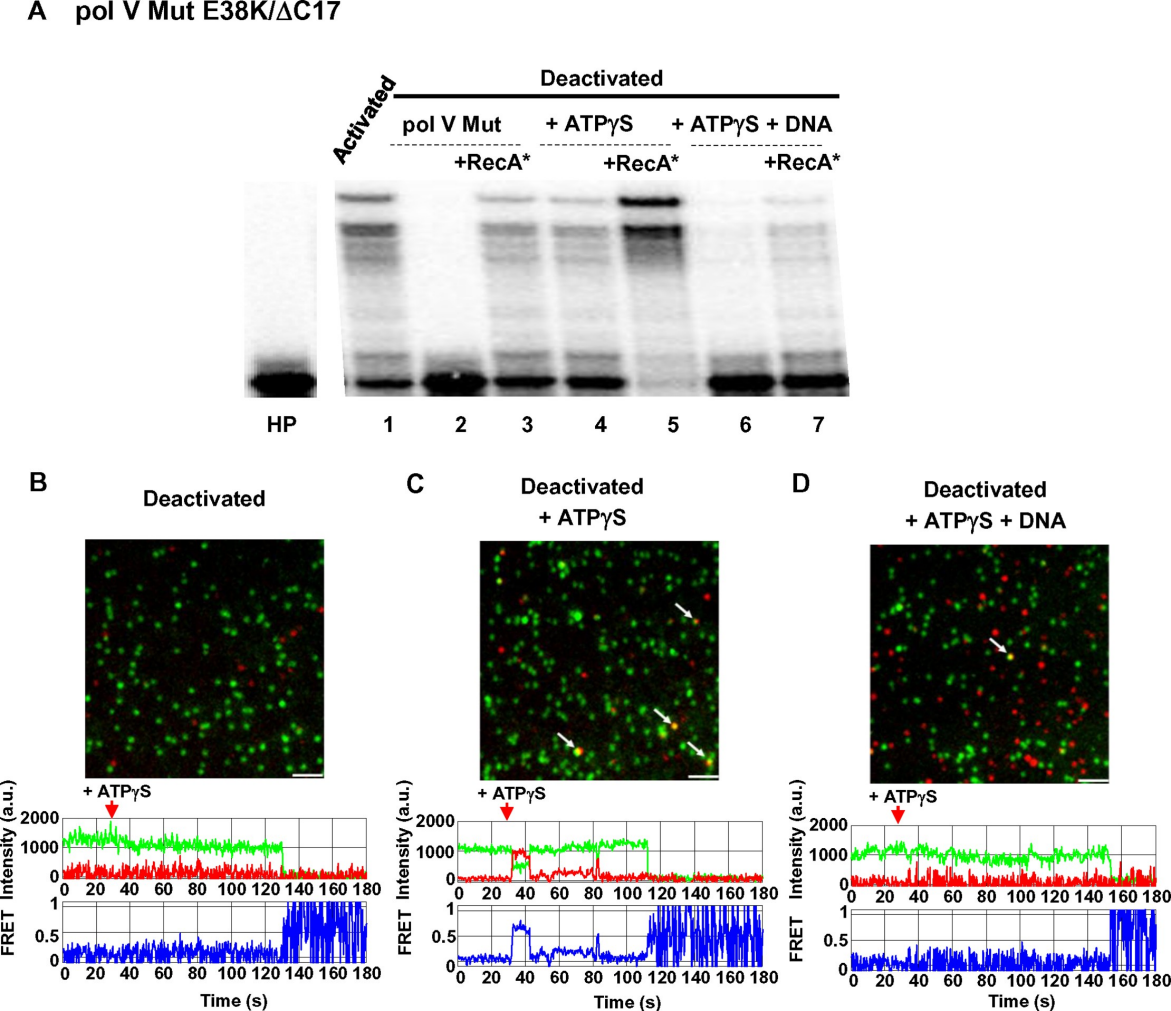
Deactivated pol V Mut E38K/ΔC17 does not bind to p/t DNA. (A) Pol V Mut was assembled using AF647-labeled RecA E38K/ΔC17. To deactivate pol V Mut_AF647_E38K/ΔC17, 1 μM of enzyme was incubated either alone (lane 2), or with ATPγS (500 μM) (lane 4), or with ATPγS (500 μM) and p/t DNA (5 μM) (lane 6) for 4 hr at 37 ^o^C. After incubation, the activity of pol V Mut E38K/ΔC17_AF647_ was measured on ^32^P-labeled 12 nt oh HP DNA in the presence of ATPγS (A), and binding to p/t DNA was detected in smFRET experiments (B-D). Statically deactivated pol V Mut E38K/ΔC17_AF647_ cannot incorporate dNTPs after 4 hr incubation at 37°C (A, lane 2). ATPγS stabilizes pol V Mut E38K/ΔC17_AF647_ and protects it from deactivating (A, lane 4). However, deactivation does occur following the addition of DNA to ATPγS-bound pol V Mut_AF647_E38K/ΔC17 (A, panel 6). Deactivated pol V Mut E38K/ΔC17_AF647_ can be reactivated by RecA* (lanes 3, 5, 7). Pol V Mut E38K/ΔC17_AF647_ that has not been subject to incubation at 37°C (4 hr incubation on ice) is able to synthesize DNA in the presence of ATPγS (lane 1). (B-D) Representative smFRET images depicting binding of deactivated pol V Mut E38K/ΔC17_AF647_ to p/t DNA. AF555-labeled p/t DNA is shown in green, pol V Mut E38K/ΔC17_AF647_ is shown in red. Binding events are shown as colocalized pol V Mut E38K/ΔC17_AF647_ and p/t DNA yellow/orange signals and are marked with arrows. Representative smFRET trajectories are shown below each deactivation panel (B-D). No DNA binding events are detected for deactivated pol V Mut E38K/ΔC17_AF647_ (B), which is consistent with the absence of polymerase activity (A, lane 2). Pol V Mut E38K/ΔC17_AF647_ activity is stabilized substantially in the presence of ATPγS, which results in DNA binding (C) and polymerase activity (A, lane 4). Fewer binding events are detected when pol V Mut E38K/ΔC17_AF647_ is incubated in the presence of ATPγS + p/t DNA (D) compared to incubation with ATPγS in the absence of DNA (B, middle panel). For all smFRET binding assays, ATPγS (500 μM) was added prior to injection of labeled pol V Mut. The images shown in (B-D) panels correspond to smFRET data integrated over 1 min. Scale bar is 150 mm.

To establish that pol V Mut can bind to p/t DNA only when activated, we measured binding using smFRET for the deactivated form of pol V Mut E38K/ΔC17 (Fig 8). Pol V Mut was completely deactivated by incubation at 37°C for 4 h in the absence of ATPγS and DNA (Fig 4A). Pol V Mut E38K/ΔC17 is active prior to incubation (Fig 8A, lane 1, t = 0). Incubation of pol V Mut E38K/ΔC17 for 4 h results in enzyme deactivation (Fig 8A, lane 2). The deactivated enzyme fails to FRET with p/t DNA even after addition of ATPγS, indicative of no binding (Fig 8B). When incubated for 4 h in the presence of ATPγS, pol V Mut E38K/ΔC17 retains substantial activity and its ability to bind DNA as observed by FRET (Fig 8A, lane 4 and Fig 8C). However, when incubated for 4 h in the presence of ATPγS + p/t DNA (in the absence of dNTPs), pol V Mut E38K/ΔC17 exhibits strong deactivation and minimal binding to p/t DNA (Fig 8A, lane 6 and Fig 8D). All deactivated forms of the polymerase can be reactivated in the presence of RecA* (Fig 8A, lanes 3, 5, and 7). The important conclusion is that statically deactivated pol V Mut cannot bind to p/t DNA.

**Fig 9:**
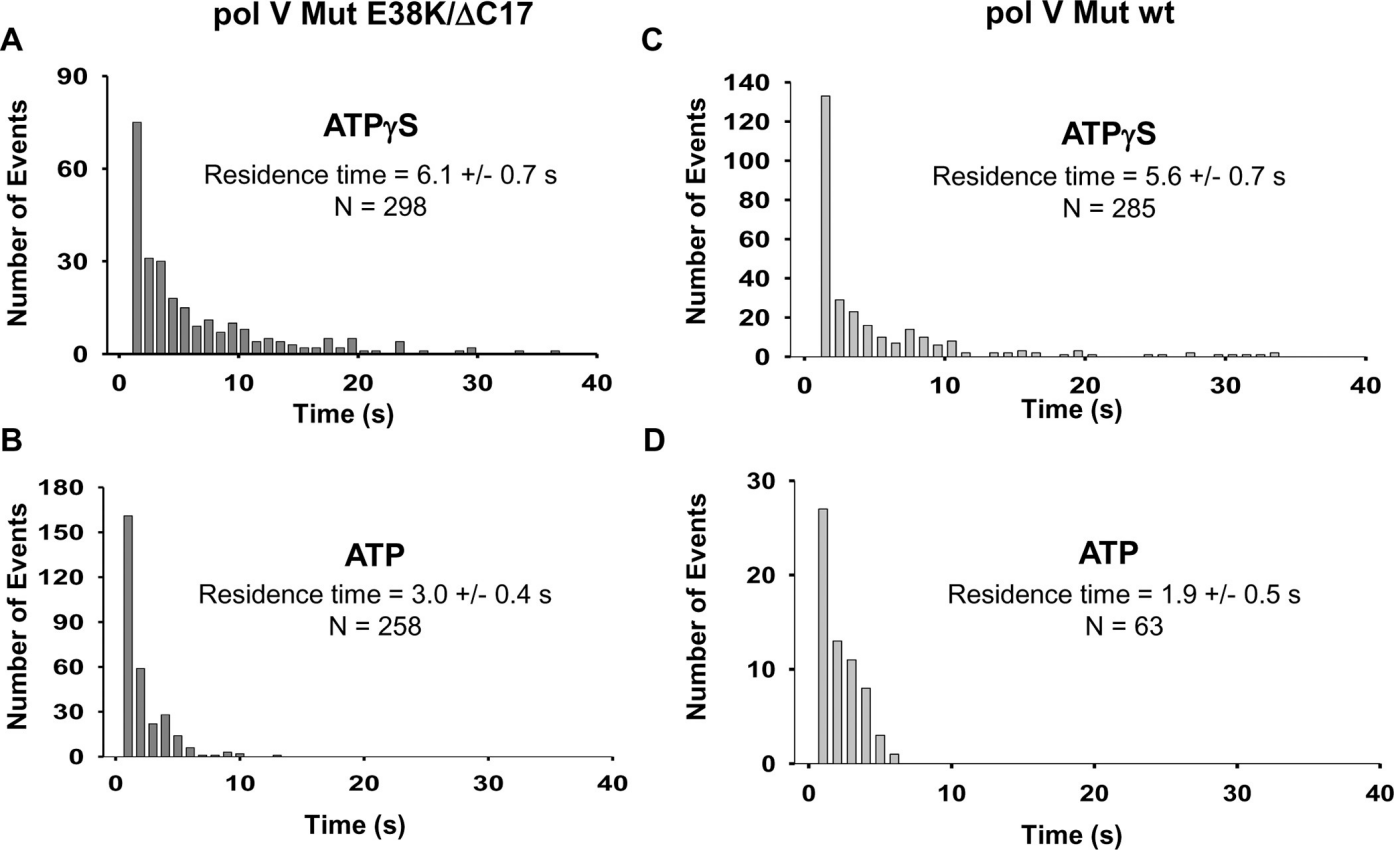
Histograms for DNA binding residence times of activated pol V Mut. Histograms representing DNA residence times of ATPγS/ATP-activated 1 nM pol V Mut E38K/ΔC17 (A-B) and 5 nM pol V Mut wt (C-D). Pol V Mut residence times were calculated by fitting the data to a single exponential decay function.

From a statistical analysis of the smFRET data with activated pol V Mut, we extracted the characteristic dwell time of binding (*τ*_bound_) of pol V Mut E38K/ΔC17 to DNA, with ATPγS or ATP (Fig 9). *τ*_bound_ is directly related to the unimolecular dissociation rate constant *k*_dissoc_ = 1/τ_bound_. Dissociation kinetics of Pol V Mut follow Poisson statistics with a typical exponential dwell time distribution. From a sample of size *N* = 298 pol V Mut E38K/ΔC17 with ATPγS, a maximum likelihood analysis [20] of the bound times yields *τ*_bound_ = 6.1 ± 0.7 s (Fig 9A). With ATP, the pol V Mut E38K/ΔC17 residence times on DNA are shifted to shorter durations (Fig 9B) with a maximum likelihood estimate giving *τ*_bound_ = 3.0 ± 0.4 s (*N* = 258). Thus, pol V Mut E38K/ΔC17 stays bound to DNA about 2 times longer with ATPγS (~6s) compared to ATP (~3s). Although this two-fold difference may seem relatively insignificant, it is important to note that the residence times follow an exponential decay. As seen in Fig 9A and Fig 9B, about 25% of the binding events persist for at least 10 s with ATPγS compared to only 1% for ATP. The longer binding times with ATPγS are reflected by increased processivities at 37°C and 30°C compared to ATP (Fig 1A and 1B). The DNA synthesis rates are about 2-fold higher for ATPγS compared to ATP, and the rates are ~ 2-fold higher at 30°C compared to 37°C (Fig 1A and 1B and S2A and S2B Fig).

With ATPγS, the dwell time of pol V Mut wt on DNA is comparable to pol V Mut E38K/ΔC17 (5.6 s versus 6.1s, Fig 9C). In contrast, with ATP, pol V Mut wt shows only a small extent of steady-state binding to DNA (Fig 3A), and there is no detectable DNA synthesis (Fig 2D and Fig 3B) [13]. The smFRET data show the presence of relatively infrequent binding events with a mean dwell time ~ < 2 s (Fig 9D). A large reduction in the numbers of binding events per unit time and shorter dwell times may account for the absence of detectible DNA synthesis with ATP (Fig 2D and Fig 3B).

The affinity constant (K_D_) of pol V Mut towards DNA can be estimated from the ratio of its dissociation rate constant *k*_dissoc_ and its association rate constant *k*_assoc_ (K_D_ = *k*_dissoc_/*k*_assoc_) where *k*_assoc_ depends on the characteristic time during which DNA is unbound (*τ*_unbound_) and the concentration of Pol V Mut (*k*_*assoc*_ = 1/(*τ*_unbound_ × [Pol V mut]). Unlike *τ*_bound_, however, *τ*_unbound_ is more difficult to establish because unbound events do not register any FRET signature. We have used a distribution of the second binding times, *τ*_rebind_, to try to estimate the relative values of *τ*_unbound_ in ATPγS versus ATP. Analysis of the smFRET data yields *τ*_rebind_ = 17 ± 2 s for 2 nM pol V Mut E38K/ΔC17 in ATPγS (*N* = 218), and 13 ± 2 s for 5 nM pol V Mut E38K/ΔC17 in ATP (*N* = 122). Together, these FRET statistics suggests that pol V Mut E38K/ΔC17 binding to DNA is approximately 3.8 times stronger in the presence of ATPγS compared to ATP.

## Discussion

The remarkably complex 4-stage regulation of pol V Mut, temporal, conformational, internal, spatial, suggests that *E*. *coli* prefers to keep this highly error-prone TLS polymerase on the sidelines to protect against chromosomal mutations on undamaged DNA templates. Proof for this point is that the SOS constitutively induced mutant, pol V Mut E38K, generates ~ 100-fold increase in spontaneous mutations in the absence of external stressors, UV or chemicals [17]. Temporal regulation, which involves the ~ 45 min delayed accumulation of pol V after SOS is turned on [2–4, 10], and spatial regulation, which involves the initial delayed synthesis and subsequent sequestering of insoluble UmuC on the cell membrane until it binds to UmuD′_2_ and is released in the cytosol [4, 21–23], are reasonably well understood. In contrast, the molecular bases for conformational and internal regulation are poorly characterized. Conformational regulation refers to the reorientation of RecA relative to UmuC [11, 12] that determines if pol V Mut is *on* or *off*. Internal regulation refers to the roles of ATP and ATP hydrolysis in affecting polymerase activity [13].

Important new insights into the mechanisms responsible for pol V Mut conformational and internal regulation can be obtained by integrating data obtained from the three sets of experiments presented here: 1) dynamic deactivation; 2) static deactivation; 3) smTIRF-FRET visualization of pol V Mut-p/t DNA binding. Taken together, these data show that pol V Mut is regulated as a conformational switch that requires the presence of RecA and ATP or ATPγS in the mutasome complex. Binding of ATP/ATPγS is required for activation (Fig 3B), and is accompanied by reorientation of RecA on pol V Mut, as shown by crosslinks formed between RecA and UmuC that require the presence of either ATP or ATPγS (Fig 6). Deactivation occurs spontaneously, and depends on time and temperature, occurring much more rapidly at 37°C than at 30°C (Figs 1, 2, 4 and 5). The rate of dynamic deactivation remains the same when more processive DNA synthesis is occurring in the presence of the β sliding clamp (S4 Fig).

**Fig 10:**
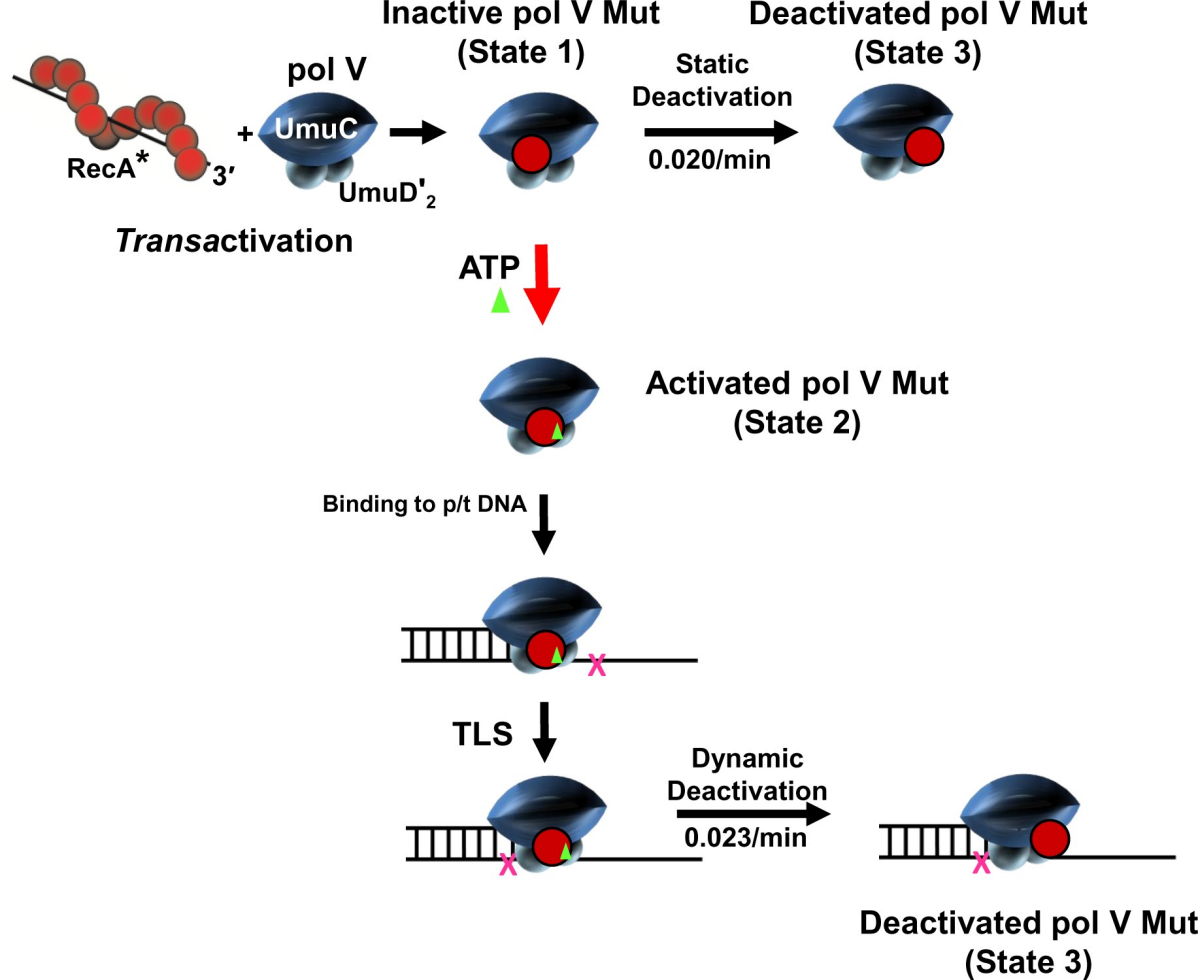
Conformational regulation of pol V Mut. The first step in pol V Mut regulatory pathway requires transfer of a molecule of RecA from the 3'-tip of a RecA* nucleoprotein filament to pol V (UmuD'_2_C) to form pol V Mut (UmuD'_2_C-RecA). Pol V Mut exists in 3 conformational states determined by the orientation of RecA on pol V. When first assembled pol V Mut (UmuD'_2_C-RecA) is catalytically inactive (State 1) and cannot bind to p/t DNA. A subsequent addition of ATP (or ATPγS) activates pol V Mut = UmuD'_2_C-RecA-ATP (State 2) due to ATP induced reorientation of RecA relative to UmuC, termed “ATP induced RecA switch”. Once activated, pol V Mut can bind to p/t DNA and synthesize DNA, including TLS (X denotes a DNA template lesion). Pol V Mut can either statically deactivate in the absence of DNA synthesis (State 3) with a rate of about 0.02/min at 37°C or deactivate dynamically (State 3) in the presence of DNA synthesis (0.023/min at 37°C). Deactivated pol V Mut can be activated (i.e., reactivated) via transfer of a “new” molecule of RecA from the 3'-tip of a RecA* nucleoprotein filament, in which the new RecA replaces the old RecA [11], followed by binding of ATP/ATPγS. Deactivated pol V Mut cannot be reactivated by binding to ATP or ATPγS. Both deactivation processes occur more rapidly at 37°C compared to at 30°C. Deactivation is inhibited in the presence of bound ATP and accelerated in the presence of bound p/t DNA – see text.

Pol V Mut exists in three separate states; catalytically inactive State 1, catalytically active State 2, catalytically deactivated State 3 (Fig 10). State 1 is formed by converting pol V (UmuD′_2_C) to pol V Mut (UmuD′_2_C-RecA), which requires a RecA*-mediated transfer of a molecule of RecA from its 3′-proximal tip to pol V. Binding of molecule of ATP or ATPγS is required as an *off* → *on* switch that converts pol V Mut from a catalytically inactive form lacking ATP/ATPγS (State 1), to the catalytically active form of pol V Mut (UmuD′_2_C-RecA-ATP) (State 2). Pol V Mut deactivates (State 3) either statically in the absence of DNA synthesis (State 1 → State 3), or dynamically during DNA synthesis (State 2 → State 3).

The two catalytically dead states (State 1 and State 3) are mechanistically distinct. Unlike State 1, the binding of ATP/ATPγS to deactivated pol V Mut (State 3) does not turn on polymerase activity, presumably because it cannot bind to p/t DNA (Fig 8B), despite the retention of a molecule of RecA in the pol V Mut complex following either static or dynamic deactivation. Deactivated pol V Mut can be reactivated via transfer of a new molecule of RecA from the 3'-tip of a RecA* nucleoprotein filament, in which the new RecA replaces the old RecA [11], followed by binding of ATP/ATPγS. We have illustrated this three-state model by placing RecA in different positions relative to UmuC (Fig 10). Repositioning of RecA on pol V Mut for active and inactive states is supported by crosslinking (Fig 6) and tandem mass spectroscopy data [12]. The N113 residue of RecA is located on a surface of the RecA nucleoprotein filament that interacts directly with pol V during assembly of pol V Mut complex (Fig 10, *Trans*activation). Early genetic studies showed that an S117F mutation eliminates UV-induced mutagenesis [24], and a subsequent biochemical study showed that pol V Mut S117F is catalytically dead [12, 19]. Mutations at nearby residues strongly reduce pol V Mut activity [12]. Using tandem mass spectrometry, we have shown that N113 of RecA is bound to the _257_ITDYPS_262_ surface on UmuC in the activated form of pol V Mut, and in an inactive state N113 is bound to UmuC at residues R_367_S_370_Q_372_ [12]. A definitive test of the model will probably require high-resolution Cryo-EM imaging.

### Pol V Mut dynamic conformational switch mechanisms regulate DNA synthesis

When carrying out DNA synthesis at 37°C with ATPγS, pol V Mut E38K/ΔC17 (Fig 1B) and pol V Mut wt (Fig 2A) deactivate dynamically in about 1.5–2 h, which is sufficient to complete just one round of synthesis (Fig 1F and Fig 2C). The dynamic deactivation rates are similar for the mutant and wild type polymerases, *D* ~ 0.02 and 0.015 min^-1^ for pol V Mut E38K/ΔC17 and pol V Mut wt, respectively (S3 Fig). However, for synthesis at 30°C, both polymerase forms deactivate much more slowly, in about 3 h, which allows for multiple template cycling, 4 rounds of synthesis for pol V Mut E38K/ΔC17 (*D* ~ 0.001 min^-1^) (Fig 1F and S3B Fig) and 3 rounds of synthesis for pol V Mut wt (*D* ~ 0.003 min^-1^) (Fig 2C, S3F Fig).

How does internal regulation involving the roles of ATP and ATP hydrolysis contribute to the mechanisms governing pol V Mut DNA synthesis? Clearly, pol V Mut’s intrinsic DNA-dependent ATPase [13] is not involved in *on* → *off* switching, since rapid dynamic deactivation occurs on a 1–3 h time scale with ATPγS (Fig 1F and Fig 2C). However, ATP binding to pol V Mut appears to provide the initial *on* → *off* regulatory switch. The presence of bound ATP is an absolute requirement for pol V Mut activity. A previous bulk biochemical analysis using rotational anisotropy to detect pol V Mut binding to fluorescein-labeled p/t DNA showed that DNA binding to a 3′-primer OH required the presence of ATP in the polymerase (ATP hydrolysis released pol V Mut from the DNA) [13]. Although steady-state rotational anisotropy is an exceptionally useful way to measure protein-DNA binding interactions, it is somewhat of a blunt tool since it cannot detect transient binding events.

The smFRET measurements visualize pol V Mut-p/t DNA binding events at sm resolution (Figs 7–9). The data demonstrate two key fundamental points. The presence of ATPγS or ATP in the pol V Mut complex is required for binding to DNA, and binding occurs only with Activated pol V Mut (Fig 10, State 2), even though RecA is present in the mutasome complex in Inactive and Deactivated states (Fig 10, State 1 and State 2, respectively).

### Biological relevance of Pol V Mut conformational regulation

In this study, we have investigated RecA-ATP/ATPγS conformational regulatory mechanisms required for pol V Mut-p/t DNA binding and DNA synthesis (activation), and cessation of synthesis (deactivation). The regulation of activation would provide a cellular mechanism to call upon pol V Mut when needed to copy damaged DNA templates. The regulation of deactivation could provide a way to limit the extent of low fidelity DNA synthesis on undamaged DNA.

It is clear that *E*. *coli* has gone to great lengths to limit the activity of error-prone pol V. This includes tight transcriptional control, inefficient post-translational processing of UmuD to UmuD’, active proteolysis of UmuD, UmuD’ and UmuC, the requirement for physical protein-protein interactions (RecA and β clamp), as well as intracellular spatial regulation (all recently reviewed in [25]). The static and dynamic deactivation mechanisms reported here provide a way to limit the opportunity for pol V to introduce adventitious mutations on undamaged DNA templates following TLS (dynamic deactivation) or from binding to undamaged DNA when diffusing free in solution (static deactivation).

A longstanding question concerns the cellular location of a RecA* nucleoprotein filament required for assembly of pol V Mut. During TLS, RecA* could be located either in *trans*, away from a template lesion, or in *cis*, on the DNA template strand immediately downstream from a lesion. Pol V can be activated in *trans* [19], as has generally been done in the experiments reported here. A *cis* arrangement has the inherent problem that a RecA* filament nucleated in a single strand DNA gap will quickly grow to encompass and thus block the adjacent 3′-primer terminus, preventing pol V binding. UmuD′_2_C can bind to an unblocked p/t DNA, but it cannot synthesize DNA until it is activated [13, 15]. Once assembled in the cell, pol V Mut is presumably stable, i.e., no longer subject to the relatively rapid proteolytic degradation of its individual UmuC and UmuD′ subunits [10]. In its activated state, containing ATP (Fig 10, State 2), pol V Mut would be able to diffuse throughout the cell to catalyze TLS causing mutations at damaged template sites, or causing adventitious mutations at undamaged template site. We envision that the conformation regulation described in our manuscript is responsible for confining mutations to sites of TLS, and to minimize the possibility of causing mutations elsewhere. It would seemingly make sense for the polymerase to retain activity for 1 to 2 hours, giving it ample time to gain access to damaged template sites and then to catalyze TLS. We further envision that following deactivation, which can occur dynamically following TLS or statically in the absence of DNA synthesis (Fig 10, State 3), that pol V remains in deactivated State 3 in the cell, unable to bind to DNA (Fig 8).

Genetic data indicate that a *trans*-RecA* pathway functions under at least some situations *in vivo*. RecA E38K mutants are constitutively induced for SOS induction and mutagenesis in the absence of externally generated DNA template damage [17]. Our biochemical data show that pol V Mut assembled with RecA wt, RecA E38K and RecA E38K/ΔC17 exhibit similar conformational regulation behavior (Figs 1, 2, 4 and 5) [11]. Recent live-cell imaging studies in a RecA E38K genetic background showed that pol V is present in high abundance in the cytosol in the absence of UV [4]. The ~ 100-fold increase in pol V-induced SOS mutations suggests that these are non-TLS mutations caused by error-prone pol V Mut E38K acting on undamaged DNA, perhaps by displacement of pol III core [26]. RecA E38K* might be formed on chromosomal DNA undergoing replication, or perhaps elsewhere in the cell. However, it is unlikely to assemble at a TLS site since few such sites would normally be available. Therefore, we suggest that for at least in the case of untargeted mutagenesis, pol V Mut E38K is likely to diffuse freely throughout the cell, as has been observed for all other DNA polymerases, and could also hold as well for damaged-induced TLS in a RecA wt genetic background.

The dynamic deactivation kinetic profiles show closely similar mechanistic properties for pol V Mut E38K/ΔC17 and pol V Mut wt when activated with ATPγS. Pol V Mut E38K/ΔC17 behaves dynamically the same with ATPγS and ATP. However, that is not the case with pol V Mut wt, which shows no measurable DNA synthesis with ATP (Fig 2B, Fig 3B). A likely explanation for the absence of detectible DNA synthesis with ATP is based on smFRET data showing a low frequency of pol V Mut-p/t DNA binding events combined with extremely short average binding times (~ 1.9 s) (Fig 8D). The presence of the β-sliding clamp is required to observe ATP-activated pol V Mut RecA wt DNA synthesis [13]. Pol V Mut contains an intrinsic DNA dependent ATPase [13]. A regulatory role for the ATPase remains an open question. Hydrolysis of a single ATP has been shown to release pol V Mut from a 3′-primer end [13]. Perhaps, the DNA-dependent ATPase acts to destabilize the pol V Mut-β clamp interaction to facilitate replication restart with pol III.

Looking ahead, a more extensive smFRET study that models DNA repair gaps, particularly for the case of pol V Mut RecA wt could include RecA* nucleoprotein filament formation within lagging-strand gaps in the presence of RecFOR, and SSB. A role for SSB may be especially germane because of a long-standing observation of a UV-nonmutable SSB mutant [27]. Mutations occur with about equal frequencies on leading- and lagging DNA strands when TLS is targeted to UV damaged DNA template sites [28]. In contrast, the lion’s share of pol V Mut-induced SOS mutations (~ 85%) occur on the lagging-strand during log-phase bacterial cell growth in the absence of external DNA damaging agents [29]. A high-resolution microscopy analysis of leading-strand continuous vs lagging-strand discontinuous gap synthesis could provide new mechanistic insights into how pol V-generated lesion-targeted vs untargeted mutations occur, which might finally reveal the mutagenic mechanism for the historically influential SOS constitutively induced RecA E38K mutant, which exhibits ~ 100-fold increase in pol V mutations in the absence of UV radiation [16].

## Methods

### Pol V Mut assembly

Pol V Mut was assembled following protocol by Erdem *et al*. 2014 [13]. Briefly, RecA nucleoprotein filament (RecA*) was assembled on Cyanogen Bromide Sepharose with covalently attached 45 nt ssDNA (3'-tip exposed). When RecA* formed, any access of unbound RecA and ATPγS was removed by extensive washes on small spin columns. His-tagged pol V (purified according to [30]) was incubated with RecA* and pol V Mut (UmuC-UmuD'_2_-RecA) complex was assembled. Pol V Mut is catalytically inactive and needs to be activated for DNA synthesis by adding ATP/ATPγS. The concentration of pol V Mut was determined by SDS-PAGE using purified pol V and RecA as protein concentration standards.

### Cycling and dynamic deactivation of pol V Mut

The activity of pol V Mut E38K/ΔC17 or pol V Mut wt was measured at 37°C and 30°C on 12 nt oh (overhang) hairpin (HP) DNA (5′-CGA AAC AGG AAA GCA GTT AGC GCA TTC AGC TCA TAC TGC TGA ATG CGC TAA CTG C-3′) in the presence of ATP/ATPγS (500 μM) and dNTP substrates (mix of dTTP, dGTP, dCTP, 500 μM each) in standard reaction buffer containing 20 mM Tris (pH 7.5), 8 mM MgCl_2_, 5 mM DTT, 0.1 mM EDTA, 25 mM sodium glutamate and 4% (v/v) glycerol. To measure the number of DNA synthesis cycles, a 5-molar access of p/t DNA (1000 nM)/pol V Mut (200 nM) was used; the 12 nt overhang (oh) HP p/t DNA contained 100 nM 5'-^32^P labeled + 900 nM unlabeled DNA strands. The DNA synthesis reactions were initiated by adding pol V Mut with ATP or ATPγS, and the reactions were carried out for up to 300 min. The deactivated pol V Mut was reactivated by adding pre-assembled 200 nM RecA*. Aliquots were removed from reactions at given time points and the reactions were terminated with a stop solution containing 20 mM EDTA in 95% formamide. The p/t DNA product molecules were separated using 20% denaturing PAGE. Gel band intensities were measured by phosphorimaging with ImageQuant software, and the fraction of extended primer (% PE) was calculated by integrating the band intensities of extended hairpin DNA divided by the total integrated DNA band intensity. Each experiment was repeated 3 times, and the average % PE (percent p/t DNA extended) with the SD (standard deviation) was plotted for each reaction time point.

The influence of the β sliding processivity clamp on pol V Mut E38K/ΔC17 deactivation was measured at 37°C on ^32^P –labeled 50 nt oh primer/template (p/t) DNA. The p/t DNA is: primer: 5'- (Biotin)dT CGA GGA TGG ATA TGG TTT AGT GGA TTT GGA TGA AGG TGA -3', template: 5' A(Biotin)dTG ACA AGA CAA GAC AAG ACA AGA CAA GAC AAG ACA AGA CAA GAC AAG AAA TCA CCT TCA TCC AAA TCC ACT AAA CCA TA -3'. Streptavidin (400 nM) was attached at both ends of the p/t DNA (25 nM) to block the β clamp from sliding off. The γ clamp-loading complex (50 nM) and ATPγS (1 mM) was used to load β (200 nM) onto the p/t DNA, and pol V Mut E38K/ΔC17 (100 nM) activity was measured for 3 h in the presence of dNTP substrates containing dTTP, dGTP and dCTP (500 μM for each substrate). In parallel, pol V Mut E38K/ΔC17 activity was measured in the absence of β clamp. Experiments for pol V Mut E38K/ΔC17 + ATPγS ± β clamp were repeated 3 times and the average % of primer extension (PE) ± SD was plotted at each reaction time.

### Calculation of rates of dynamic pol V Mut deactivation

The rate of pol V Mut primer extension follows first-order kinetics: *dP*/*dt* = *k*′*P*, where the pseudo-first-order rate constant *k*′ is proportional to the concentration of active enzyme *k*′ = *k*[*E*]. During the reaction, we assumed dynamic deactivation depletes the active enzyme concentration [*E*] by a Poisson process with deactivation rate *D*. Integrating the rate equation gives ln[1−*P*(*t*)] = −*kt*+*k*(*e*^−*Dt*^−*Dt*−1)/*D*, where *P*(*t*) is the fraction of primer extension as a function of time *t*. At short times, this equation reduces to ln[1−*P*(*t*)] = −*kt*. Fitting the initial rate to a straight line therefore yields the intrinsic catalytic rate constant *k*, and then fitting the long-time data to the full equation produces the dynamic deactivation rate *D* (S3 Fig). The parameters *k* and *D* ± SD were determined from an average of at least 3 independent measurements.

### Thermal Inactivation of pol V Mut, pol V, and RecA

Pol V Mut E38K/ΔC17 (300 nM), pol V Mut wt (300 nM), pol V (300 nM), RecA E38K/ΔC17 (9 μM) and RecA wt (9 μM) were incubated for 15 min at 37°C, 40°C, 42°C, and 45°C in standard reaction buffer (see above) followed by a measurement of DNA polymerase activity on 12 nt oh HP DNA (600 nM) at 37°C for 1h. The activity of pol V Mut E38K/ΔC17 (300 nM) and pol V Mut wt (300 nM) was measured with ATPγS (500 μM) and dNTP substrates (dTTP, dGTP, dCTP, 500 μM each). Pol V activity was measured after transactivation with RecA* (300 nM). The effect of temperature on RecA monomers was measured by their ability to form RecA* nucleoprotein filaments on 30 nt long ssDNA (600 nM) in the presence of ATPγS (500 uM) followed by *trans*activation of pol V (300 nM).

### Static deactivation of pol V Mut

Pol V Mut E38K/ΔC17 (600 nM) and pol V Mut wt (600 nM) were incubated at 37°C or 30°C in standard reaction buffer (see above), either alone or with 500 μM ATPγS/ATP, or with 500 μM ATPγS/ATP and 900 nM 12 nt oh HP DNA. Each static deactivation incubation was carried out for up to 240 min at either at 37°C or 30°C, and for each incubation time an aliquot of protein was removed and pol V Mut activity was measured at 37°C on 12 nt HP DNA (final concentration in the reaction = 500 nM) in the presence of ATPγS (final concentration = 500 μM) and dNTP substrates (mix of dTTP, dGTP, dCTP, 500 μM each) for 1 h. The DNA synthesis products were separated on a 20% denaturing polyacrylamide gel, and the bands intensities were calculated using ImageQuant. Static deactivation is expressed as the relative polymerase activity measured at each incubation time point divided by the polymerase activity measured at t = 0. Each experiment was repeated 3 times and average relative activity along with the SD for each deactivation time point are shown in the graphs.

An estimate of the initial static deactivation rate was obtained from the reduction in activity at 30 min (S5 Fig), and the difference in static deactivation rates at 37°C and 30°C were used to determine a rough estimate of the difference in activation energies between pol V Mut E38K/ΔC17 and pol V Mut wt. The activation energy was extracted from an Arrhenius analysis of the initial rates of decay in enzyme activity at the two temperatures.

### Engineering, labeling and purification of *p*Azpa substituted RecA

To site specifically label RecA E38K/ΔC17 and RecA wt for smFRET experiments we substituted F21 amino acid with *p*-azido-L-phenyloalanine (*p*Azpa). The RecA wt_F21*p*Azpa_ and RecA E38K/ΔC17_F21*p*Azpa_ was engineered, expressed, and purified as previously described [31]. To fluorescently label *p*-azido-phenylalanine modified RecA E38K/ΔC17 and RecA wt, 1 ml of 100 μM RecA wt_F21*p*Azpa_ and RecA E38K/ΔC17_F21*p*Azpa_ was incubated with Alexa Fluor 647 DIBO Alkyne (AF647; ThermoFisher Scientific) at a ratio of 1:5 in 20 mM Tris, 0.1 mM EDTA, 10% glycerol pH 7.5 buffer and rotated at 4°C overnight. Following incubation, dye and protein mixture were loaded onto a Ceramic Hydroxyapatite column (Bio-Rad) and the labeled RecA was eluted by running a phosphate gradient from 0 to 0.25 M potassium phosphate. Unbound dye eluted through the wash, AF647-labeled RecA eluted early during the potassium phosphate gradient and unlabeled RecA eluted last. Labeled RecA concentrations and labeling efficiency were determined via spectrophotometry at 280 nm and 650 nm respectively.

### Synthesis and labeling of p/t DNA for smFRET

Biotinylated 40 nt primer (5'- (Biotin)dT–CGA GGA TGG ATA TGG TTT AGT GGA TTT GGA TGA AGG TGA -3') and C6-amino-modified 54 nt long template (5′- TAG CAT GCG TCA GCT TCA CC AF555-T TCA TCC AAA TCC ACT AAA CCA TAT CCA TCC TCG -3′) were purchased from Integrated DNA Technologies. The ssDNA oligos were purified trough 20% denaturing polyacrylamide gel electrophoresis (PAGE). To label C6-amino-modified template, 100 μl of 100 μM template was incubated with Alexa Fluor 555 NHS Ester (AF555; ThermoFisher) and rotated at 4°C overnight in 0.1 M sodium bicarbonate pH 8.3. AF555-labeled template was purified trough PAGE. Prior to smFRET experiments, biotinylated DNA primer and AF555 template were annealed at a ratio of 1:1.2 by heating to 95°C and allowed to slowly cool to 16°C by decreasing 1°C per minute in a thermocycler.

### Single-molecule FRET microscopy measurements

For single-molecule FRET experiments, high precision microscope glass coverslips (Marienfeld, #1.5, Ø25 mm) were first cleaned by sonication in ddH_2_0 for 1 min followed by 100 mM KOH for 20 min. Slides were then cleaned in a Piranha solution of 1:3 hydrogen peroxide to sulfuric acid for 5 min followed by further sonication in ddH_2_0 for 10 min to clean off Piranha residue. Coverslips were then incubated in a solution containing 3 ml of 3-Aminopropyltriethoxysilane (Sigma-Aldrich), 5 ml acetic acid, and 100 ml methanol for 20 min with 1-min sonication in the middle of the incubation. The coverslips were rinsed with methanol and dried prior to applying a solution containing 25 mM of a 5:1 polyethylene glycol (PEG) and biotin-PEG-Succinimidyl Valerate (Laysan Bio) in 0.1 M sodium bicarbonate pH 8.3 buffer and incubated overnight for surface functionalization. To reduce background fluorescence [32], a second round of surface pegylation was done for 2 hr using 25 mM of 4-Methyl-PEG-NHS-Ester (Thermo-Fisher), prior to washing with ddH20 and incubation with streptavidin (Sigma) for 10 min in buffer containing 20 mM Tris-HCl, 50 mM NaCl pH 7.5. Coverslips were then washed and coated with 20 pM AF555-labeled primer template DNA for 5 min. Unbound p/t DNA was washed off in imaging buffer (1X reaction buffer + 50 ug/ml BSA, 2mM Trolox, 10 mM PCA, and 100 nM PCD [33]) prior to the addition of pol V Mut labeled with Alexa 647 at F21 position of RecA wt or RecA E38K/ΔC17. 1X reaction buffer contains 20mM Tris-HCl pH 7.5, 25 mM sodium glutamate, 8 mM MgCl_2_, 5 mM DTT, 4% glycerol, 0.1 mM EDTA.

To assess pol V Mut binding on AF555- labeled p/t DNA, pol V Mut alone (incubation 1 see Results and Fig 7D, right panel) or ATPγS activated pol V Mut (incubation 3, see Results and Fig 7D, right panel) were added at t = 30 s after start of image acquisition. For incubation 2 (pol V Mut + ATPγS; see results and Fig 7D middle panel), pol V Mut was added at t = 30 s and 500 uM ATPγS was added at t = 60 s after start of image acquisition to assess the impact of ATPγS on pol V Mut DNA binding. Each experiment was carried out for 3 min, after which donor bleaching is observed. smFRET with deactivated pol V Mut was done as follow: 1 μM pol V Mut was incubated at 37°C in 1x reaction buffer +/- ATPγS and +/- DNA for 4 h prior to adding onto AF555-labeled p/t DNA covered slides. Pol V Mut was added in the presence of 500 μM ATPγS at t = 30 s after the start of image acquisition.

Fluorescence imaging was performed on an inverted Nikon Eclipse Ti-E microscope equipped with total internal reflection optics, 561 nm and 647 nm fiber-coupled excitation lasers (Agilent), a x100 1.49 NA objective, a two-camera imaging splitter (Andor) and two iXon EMCCD cameras (Andor). A multi-band pass ZET405/488/561/647x excitation filter (Chroma), a quad-band ZT405/488/561/647 dichroic mirror (Chroma), an emission splitting FF-640-FDi01 dichroic mirror (Semrock) and two emission filters at 600/50 nm (Chroma) and 700/75 nm (Chroma) for AF555 p/t DNA and pol V Mut RecA wt_AF647_ respectively, were used. smFRET images were acquired using 561 nm laser excitation at an image acquisition rate of 100 ms/frame in each channel for ATP and 300 ms/frame for ATPγS. Channel alignment was performed using a few 40 nm TransFluoSphere beads (488/685 nm, Life Technologies) as fiducials markers.

### smFRET calculations

The 8x8 pixel region of interests were drawn around individual p/t DNA molecules in overlaid two-color images, and signal intensity from both channels were extracted for each acquisition frame. Automated detection of countercorrelated smFRET signals on the slides were performed using in-house AI software. FRET efficiency was calculated using the formula:

$$E=I_{A}/\left(I_{D}+I_{A}\right)$$

where I_D_, is the signal intensity from an individual AF555-labeled p/t DNA molecule and I_A_ is the signal intensity of a bound AF647-Pol V Mut in each frame. The smFRET efficiency histograms of Fig 9 were produced by binning the smFRET efficiency values for multiple individual smFRET pairs by 1 second. τ-on and τ-rebind were determined by measuring the residence time and time between two binding events, respectively, plotting them as a histogram and fitting the histogram with a single exponential decay function.

### UV crosslinking experiment

For UV crosslinking experiment, RecA wt and RecA E38K/ΔC17 was modified with *p*Bpa at the N113 and F21 positions and purified as previously described [12]. Pol V Mut was assembled with RecA E38K/ΔC17_N113*p*BpA_, RecA wt_N113*p*Bpa_, RecA E38K/ΔC17_F21*p*BpA_ and RecA wt_F21*p*BpA_ were incubated for 30 min at 37°C either alone or with ATP/ATPγS (500μM) in the presence of absence of 12 nt oh HP DNA. Following pre-incubation, the reactions were exposed to UV (365 nm) for 60 min, with gentle mixing at 15 min intervals. Crosslinked products were boiled in protein loading dye and separated using 12% SDS-PAGE. UmuC-RecA crosslinked bands were detected using affinity-purified rabbit anti-UmuC (1:200 dilution) or UmuD/UmuD′ (1:1000 dilution), using a standard Western Blot protocol. Each crosslinking experiment was repeated 3–4 times, and the % of crosslinked UmuC-RecA was calculated by measuring Western blot intensities with ImageQuant and ImageJ software.

## Supporting information

S1 Fig Cycling of pol V Mut E38K/ΔC17 at 30°C with 10-fold molar excess of p/t DNA. To detect dynamic deactivation of pol V Mut E38K/ΔC17 (200 nM), DNA synthesis was measured with a 10-fold excess of ^32^P-labelled 12 nt oh HP p/t DNA (2 μM) in the presence of a saturating concentration of ATPγS (500 μM) and dNTP’s (mix of dTTP, dCTP, dGTP 500 μM each). Deactivated pol V Mut is reactivated in the presence of RecA* (200 nM).
(TIF)

S2 Fig Thermal inactivation of pol V Mut, pol V and RecA. (A) Sketch showing the experimental protocol used to measure the thermal inactivation of pol V Mut, pol V, and RecA. Each protein was incubated for 15 min at 37°C, 40°C, 42°C, and 45°C followed by a measurement of DNA polymerase activity at 37°C for 1h. Pol V Mut activity (A, left reaction scheme) was measured with ATPγS and dNTPs; pol V activity (A, middle reaction scheme) was measured after *trans*activation with RecA*. To detect effect of temperature on RecA monomers, we measured their ability to form RecA* nucleoprotein filaments and *trans*activate pol V (A, right reaction scheme). Pol V Mut wt (B) and pol V Mut E38K/ΔC 17 (E) are irreversibly inactivated at 45°C. In contrast to either dynamic or static deactivated forms of pol V Mut (see Figs 1 and 2 and Figs 4 and 5), the inactive form of pol V Mut cannot be reactivated by RecA*. When incubated alone at 45°C, pol V (C) and RecA (E, F) remain functionally “active” in the sense that they retain the ability to assemble into an activated form of pol V Mut.
(TIF)

S3 Fig Analysis of dynamic deactivation profiles. (A-F) A 2-parameter fit to the dynamic deactivation profiles (Fig 1 and Fig 2) is used to calculate the rate constant for DNA synthesis (*k*) and the deactivation rate (*D*). The rate constant *k* is determined from the initial rate of primer extension as a function of time, i.e., from the early time points prior to the onset of significant deactivation (red line). The rate *D* is determined from the entire deactivation profile (yellow curve). The values of *k* (~ 0.004–0.008) at 37°C are similar with pol V Mut E38K/ΔC17 (A, C) and pol V Mut wt (E), assembled with either ATPγS or ATP; the values of *k* are about 3-fold higher at 30°C (B, D, F). The values of *D* (~ 0.015–0.028) at 37°C are also similar for both forms of pol V Mut, with ATPγS (A, E) or ATP (C); the values of *D* are about 3-fold lower at 30°C (B, D, F) compared to 37°C (A, C, E). The parameters *k* and *D* ± SD were determined from an average of at least 3 independent measurements. The calculations for *k* and *D* are described under Methods.
(TIF)

S4 Fig Dynamic deactivation of pol V Mut E38K/ΔC17 at 37°C in the absence and presence of β clamp. The dynamic deactivation of pol V Mut E38K/ΔC17 (100 nM) was measured in the absence (A) and presence (B) of the β processivity clamp, using ^32^P-labelled 50 nt oh p/t DNA (25 nM) at 37°C. Biotin-streptavidin was attached to the ends of p/t DNA to prevent the β clamp from sliding off the DNA. Pol V Mut E38K/ΔC17 activity was measured in the presence of a saturating concentration of ATPγS (1mM) and dNTP’s (mix of dTTP, dCTP, and dGTP, 500 μM each). Representative DNA synthesis gels are presented in (A) and (B). Experiments for pol V Mut E38K/ΔC17 + ATPγS ± β clamp were repeated 3 times and the average % of primer extension (PE) ± SD was plotted for each reaction time (C). A 2-parameter fit to the dynamic deactivation profiles was used to calculate the rate constant for DNA synthesis (*k*) and the deactivation rate (*D*) ± β clamp. The *k* and *D* rates are comparable in the absence (*k* = 0.012, *D* = 0.06) and in the presence of β clamp (*k* = 0.019, *D* = 0.07).
(TIF)

S5 Fig Rates of static deactivation of pol V Mut E38K/ΔC17 and pol V Mut wt. Static deactivation rates were calculated as the reduction in the relative polymerase activity at 30 min compared to 0 min at 37°C and 30°C; pol V Mut E38K/ΔC17 (A), pol V Mut wt (B). Each rate is an average of 2–3 independent measurements with SD provided. The activation energies were extracted from an Arrhenius analysis of the initial rates of decay in enzyme activity at the two temperatures, and are estimated to be 22 kcal/mol for pol V Mut E38K/ΔC17 and 9 kcal/ mol for pol V Mut wt.
(TIF)

S6 Fig Static Deactivation of pol V Mut E38K/ΔC17 at 37°C and 30°C. Relative activity of Pol V Mut E38K/ΔC17 (A and B) or pol V Mut wt (C and D) incubated from 0 to 4h at 37°C and 30°C either alone (black circles), with ATP (white circles), or with ATP + 12nt oh HP p/t DNA (black triangles). At each incubation time point, an aliquot of protein was removed, and polymerase activity was measured at 37°C for 1h on ^32^P-12 nt oh HP p/t DNA and saturating concentration of ATPγS and dNTPs (dTTP, dCTP, and dGTP). Static deactivation is slower at 30°C compared to 37°C.
(TIF)

S7 Fig Efficiency of UmuC-RecA crosslinking in the presence of ATP, ATPγS, and DNA. The % of UmuC-RecA crosslinking for (A) pol V Mut E38K/ΔC17_N113*p*Bpa_ and (B) pol V Mut wt _N113*p*Bpa_ that results from binding first to ATPγS/ATP (500μM), and then to p/t DNA DNA (5μM) in the presence of ATPγS/ATP. The data were obtained from four independent crosslinking measurements.
(TIF)

S8 Fig Pol V Mut activity and crosslinking patterns determined in the presence of ATP, ATPγS, ADP, AMP, and AMPPNP. (A) Pol V Mut E38K/ΔC17_N113pBpa_ activity with ATP, ATPγS, ADP, AMP, and AMPPNP. (B) UmuC-RecA crosslinking for pol V Mut E38K/ΔC17_N113pBpa_ with ATP, ATPγS, ADP, AMP, and AMPPNP. RecA E38K/ΔC17_N113pBpa_ forms crosslinks to UmuC in the presence of ATP, ATPγS, and ADP, but not with either AMP or AMPPNP. (C) Pol V Mut wt_N113pBpa_ activity with ATP, ATPγS, ADP, AMP, and AMPPNP. (D) UmuC-RecA crosslinking for pol V Mut wt_N113pBpa_ with ATP, ATPγS, ADP, AMP, and AMPPNP. RecA wt_N113pBpa_ forms crosslinks to UmuC in the presence of ATP, ATPγS, and ADP, but not with either AMP or AMPPNP. (E) Absence of crosslinking between RecA wt_N113pBpa_ and UmuD' for pol V Mut wt. This result was previously reported in Gruber *et*. *al* 2015 [12] and repeated here. Each crosslinking experiment was repeated 4 times.
(TIF)

S9 Fig RecA_F21*p*Bpa_ forms a crosslink with UmuD' of pol V Mut E38K/ΔC17 and pol V Mut wt. Pol V Mut was assembled with crosslinkable (A-B) RecA E38K/ΔC17_F21*p*Bpa_ and (C-D) RecA wt_F21*p*Bpa_. RecA E38K/ΔC17_F21*p*Bpa_ (A) and RecA wt_F21*p*Bpa_ (D) crosslinks to UmuD' of pol V Mut. There is no crosslinking observed between either RecA E38K/ΔC17_F21*p*Bpa_ (B) or RecA wt_F21*p*Bpa_ (D) and UmuC. Each crosslinking experiment was repeated 3 times.
(TIF)

S1 Movie smFRET imaging of pol V Mut E38K/ΔC17 binding to p/t DNA in the absence of ATPγS. Pol V Mut does not bind p/t DNA in the absence of ATPγS and no smFRET is detected. Movie acquired at 300 ms/frame and displayed at 26 ms/frame.
(MP4)

S2 Movie smFRET imaging of pol V Mut E38K/ΔC17 binding to p/t DNA after addition of ATPγS. Pol V Mut binds p/t DNA only after addition of ATPγS as indicated by the appearance of smFRET signals. Movie acquired at 300 ms/frame and displayed at 26 ms/frame.
(MP4)

S3 Movie smFRET imaging of p/t DNA binding by ATPγS-activated pol V Mut E38K/ΔC17. Pol V Mut rapidly binds to p/t DNA with smFRET events detected within a few seconds. Movie acquired at 300 ms/frame and displayed at 26 ms/frame.
(MP4)
