# Supplementary figures and images for: Conformational regulation of Escherichia coli DNA polymerase V by RecA and ATP
Source: PLoS Genet. 2019 Feb 4;15(2):e1007956. doi: 10.1371/journal.pgen.1007956 (PMC6375631; doi:10.1371/journal.pgen.1007956)

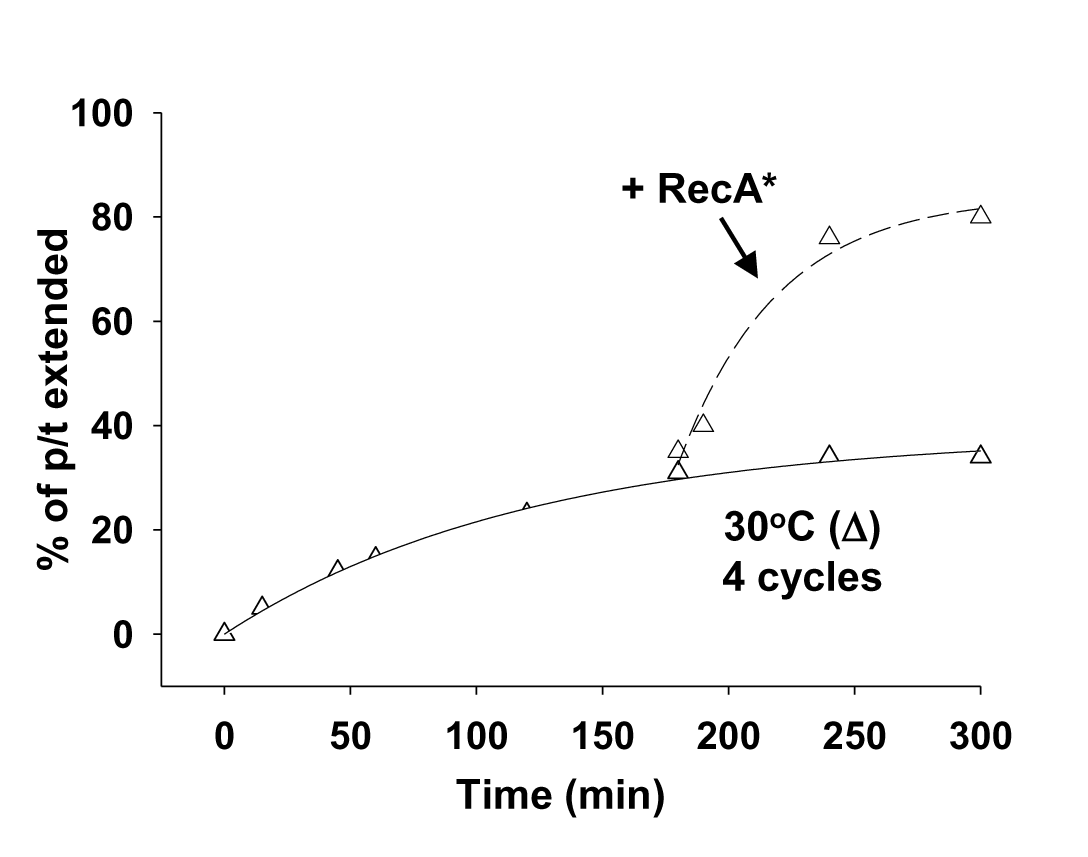

Supplement: S1 Fig — To detect dynamic deactivation of pol V Mut E38K/ΔC17 (200 nM), DNA synthesis was measured with a 10-fold excess of 32P-labelled 12 nt oh HP p/t DNA (2 μM) in the presence of a saturating concentration of ATPγS (500 μM) and dNTP’s (mix of dTTP, dCTP, dGTP 500 μM each). Deactivated pol V Mut is reactivated in the presence of RecA* (200 nM). (TIF) [file pgen.1007956.s001.tif]

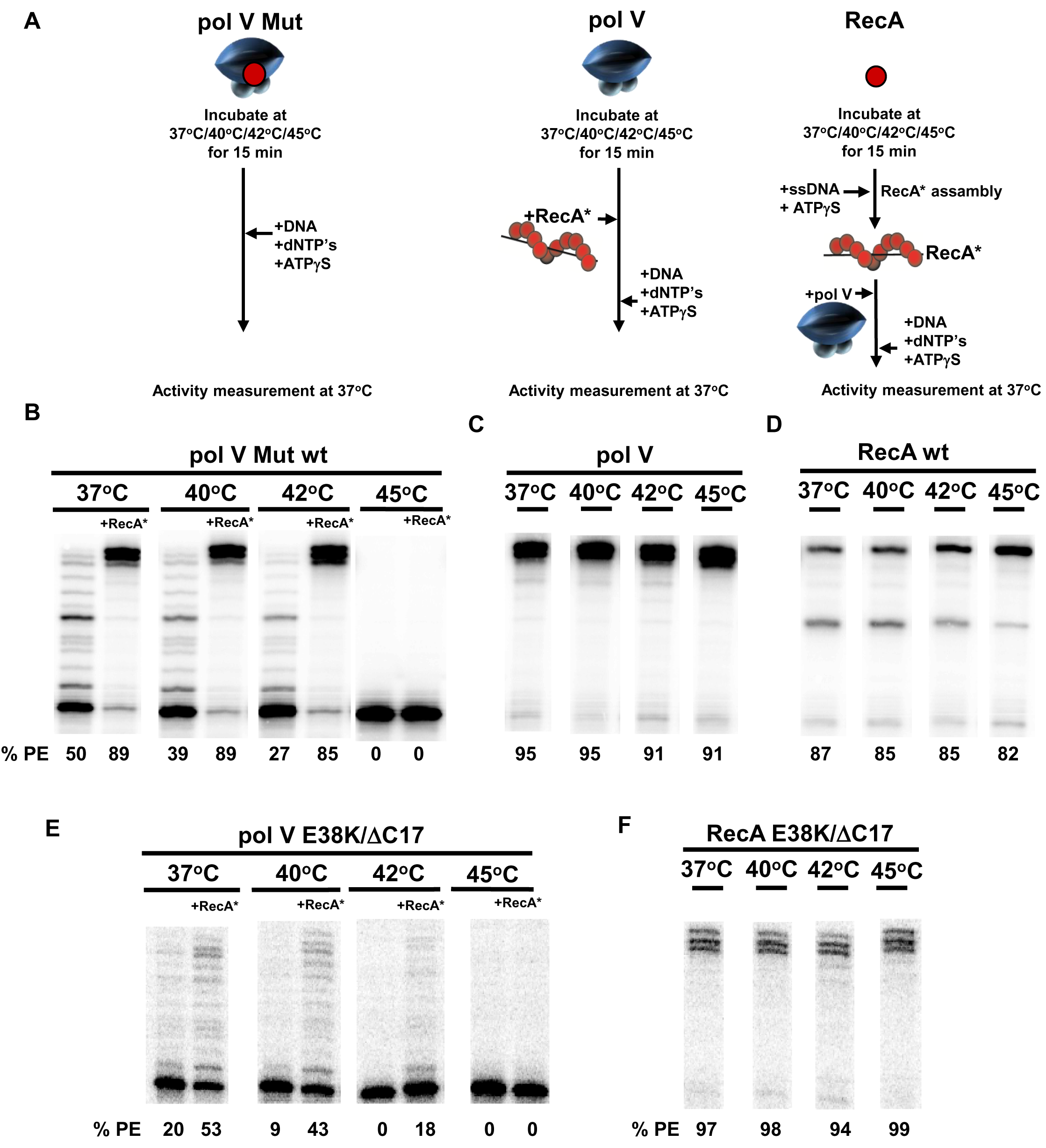

Supplement: S2 Fig — (A) Sketch showing the experimental protocol used to measure the thermal inactivation of pol V Mut, pol V, and RecA. Each protein was incubated for 15 min at 37°C, 40°C, 42°C, and 45°C followed by a measurement of DNA polymerase activity at 37°C for 1h. Pol V Mut activity (A, left reaction scheme) was measured with ATPγS and dNTPs; pol V activity (A, middle reaction scheme) was measured after transactivation with RecA*. To detect effect of temperature on RecA monomers, we measured their ability to form RecA* nucleoprotein filaments and transactivate pol V (A, right reaction scheme). Pol V Mut wt (B) and pol V Mut E38K/ΔC 17 (E) are irreversibly inactivated at 45°C. In contrast to either dynamic or static deactivated forms of pol V Mut (see Figs 1 and 2 and Figs 4 and 5), the inactive form of pol V Mut cannot be reactivated by RecA*. When incubated alone at 45°C, pol V (C) and RecA (E, F) remain functionally “active” in the sense that they retain the ability to assemble into an activated form of pol V Mut. (TIF) [file pgen.1007956.s002.tif]

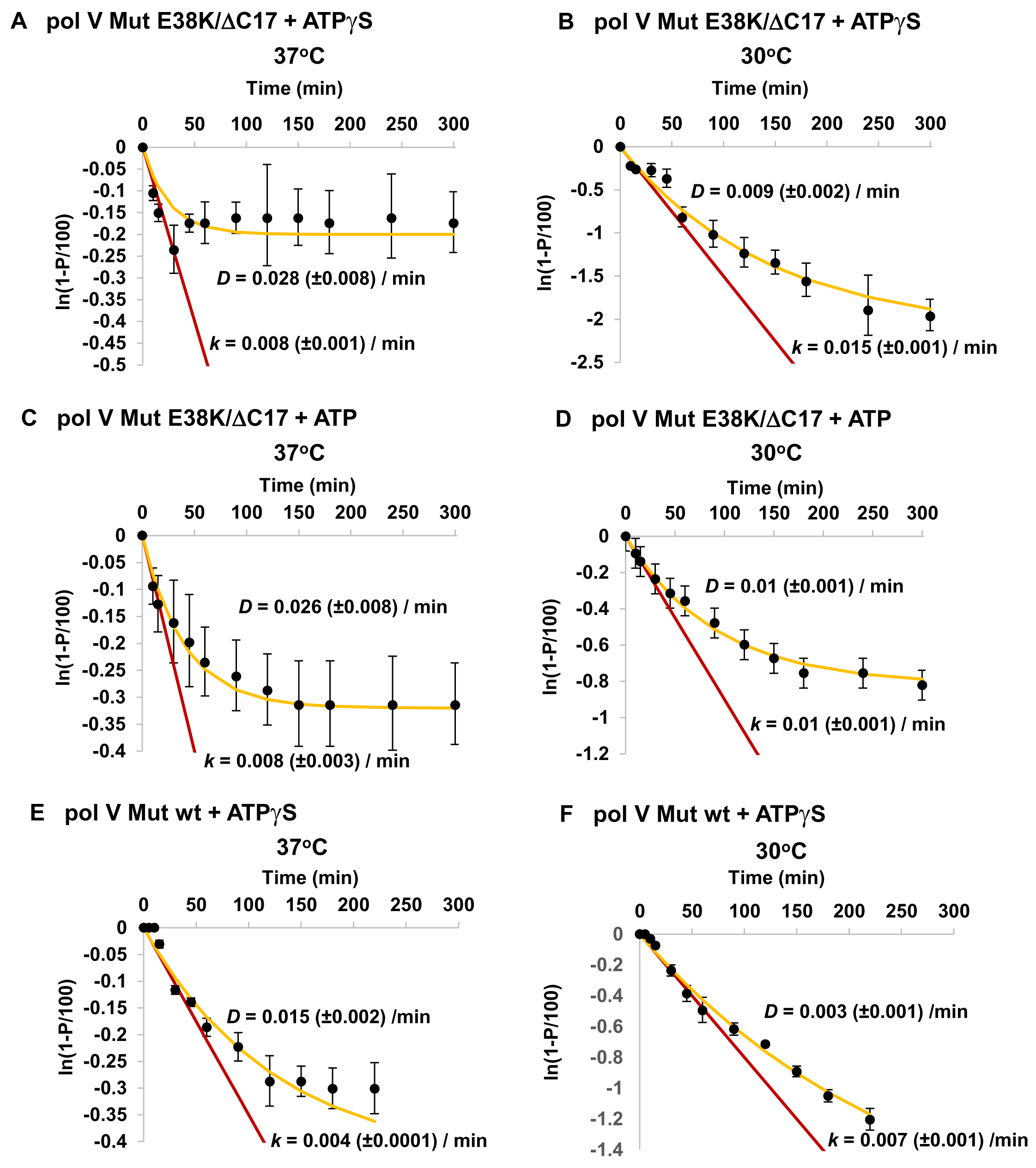

Supplement: S3 Fig — (A-F) A 2-parameter fit to the dynamic deactivation profiles (Fig 1 and Fig 2) is used to calculate the rate constant for DNA synthesis (k) and the deactivation rate (D). The rate constant k is determined from the initial rate of primer extension as a function of time, i.e., from the early time points prior to the onset of significant deactivation (red line). The rate D is determined from the entire deactivation profile (yellow curve). The values of k (~ 0.004–0.008) at 37°C are similar with pol V Mut E38K/ΔC17 (A, C) and pol V Mut wt (E), assembled with either ATPγS or ATP; the values of k are about 3-fold higher at 30°C (B, D, F). The values of D (~ 0.015–0.028) at 37°C are also similar for both forms of pol V Mut, with ATPγS (A, E) or ATP (C); the values of D are about 3-fold lower at 30°C (B, D, F) compared to 37°C (A, C, E). The parameters k and D ± SD were determined from an average of at least 3 independent measurements. The calculations for k and D are described under Methods. (TIF) [file pgen.1007956.s003.tif]

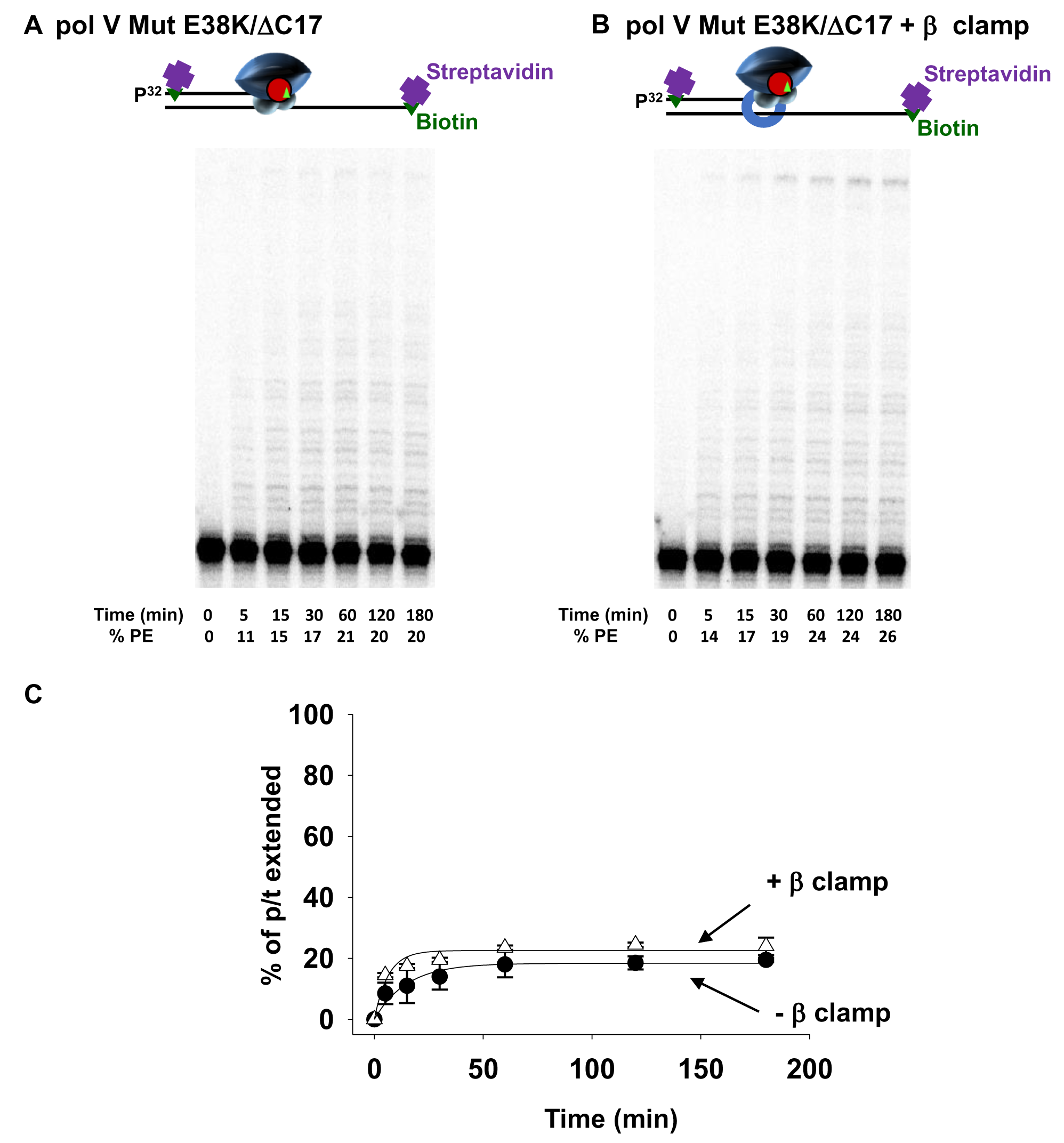

Supplement: S4 Fig — The dynamic deactivation of pol V Mut E38K/ΔC17 (100 nM) was measured in the absence (A) and presence (B) of the β processivity clamp, using 32P-labelled 50 nt oh p/t DNA (25 nM) at 37°C. Biotin-streptavidin was attached to the ends of p/t DNA to prevent the β clamp from sliding off the DNA. Pol V Mut E38K/ΔC17 activity was measured in the presence of a saturating concentration of ATPγS (1mM) and dNTP’s (mix of dTTP, dCTP, and dGTP, 500 μM each). Representative DNA synthesis gels are presented in (A) and (B). Experiments for pol V Mut E38K/ΔC17 + ATPγS ± β clamp were repeated 3 times and the average % of primer extension (PE) ± SD was plotted for each reaction time (C). A 2-parameter fit to the dynamic deactivation profiles was used to calculate the rate constant for DNA synthesis (k) and the deactivation rate (D) ± β clamp. The k and D rates are comparable in the absence (k = 0.012, D = 0.06) and in the presence of β clamp (k = 0.019, D = 0.07). (TIF) [file pgen.1007956.s004.tif]

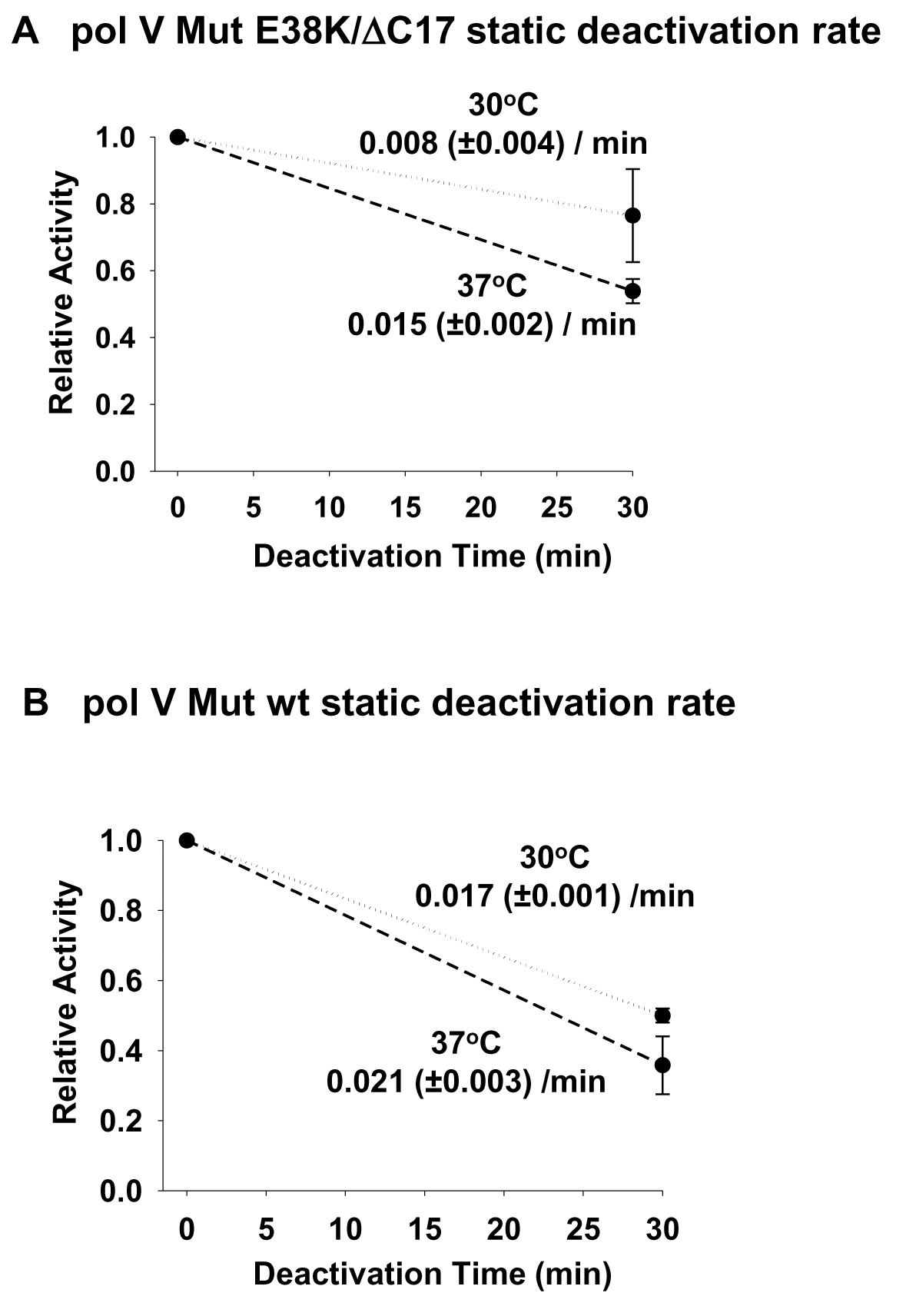

Supplement: S5 Fig — Static deactivation rates were calculated as the reduction in the relative polymerase activity at 30 min compared to 0 min at 37°C and 30°C; pol V Mut E38K/ΔC17 (A), pol V Mut wt (B). Each rate is an average of 2–3 independent measurements with SD provided. The activation energies were extracted from an Arrhenius analysis of the initial rates of decay in enzyme activity at the two temperatures, and are estimated to be 22 kcal/mol for pol V Mut E38K/ΔC17 and 9 kcal/ mol for pol V Mut wt. (TIF) [file pgen.1007956.s005.tif]

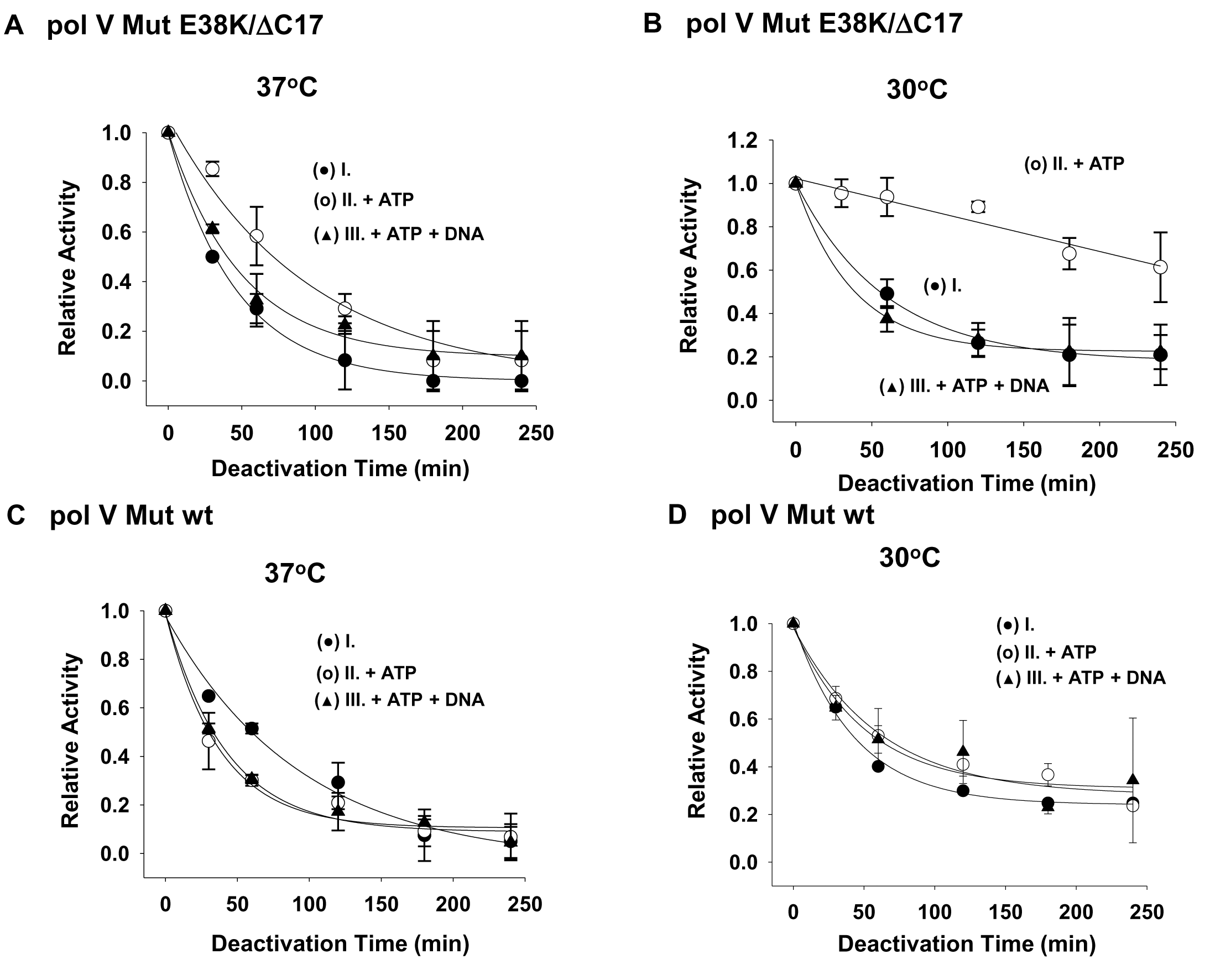

Supplement: S6 Fig — Relative activity of Pol V Mut E38K/ΔC17 (A and B) or pol V Mut wt (C and D) incubated from 0 to 4h at 37°C and 30°C either alone (black circles), with ATP (white circles), or with ATP + 12nt oh HP p/t DNA (black triangles). At each incubation time point, an aliquot of protein was removed, and polymerase activity was measured at 37°C for 1h on 32P-12 nt oh HP p/t DNA and saturating concentration of ATPγS and dNTPs (dTTP, dCTP, and dGTP). Static deactivation is slower at 30°C compared to 37°C. (TIF) [file pgen.1007956.s006.tif]

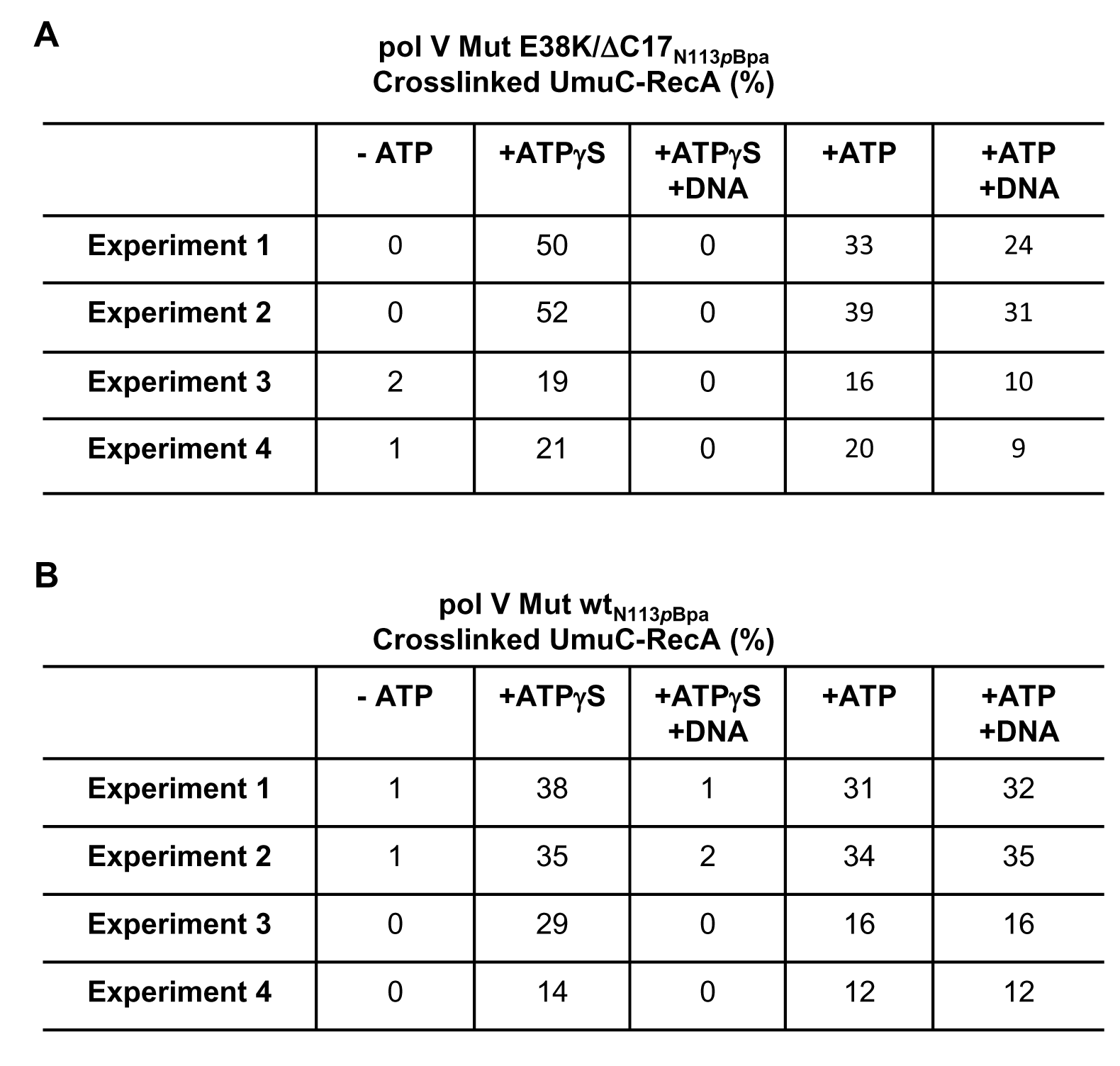

Supplement: S7 Fig — The % of UmuC-RecA crosslinking for (A) pol V Mut E38K/ΔC17N113pBpa and (B) pol V Mut wt N113pBpa that results from binding first to ATPγS/ATP (500μM), and then to p/t DNA DNA (5μM) in the presence of ATPγS/ATP. The data were obtained from four independent crosslinking measurements. (TIF) [file pgen.1007956.s007.tif]

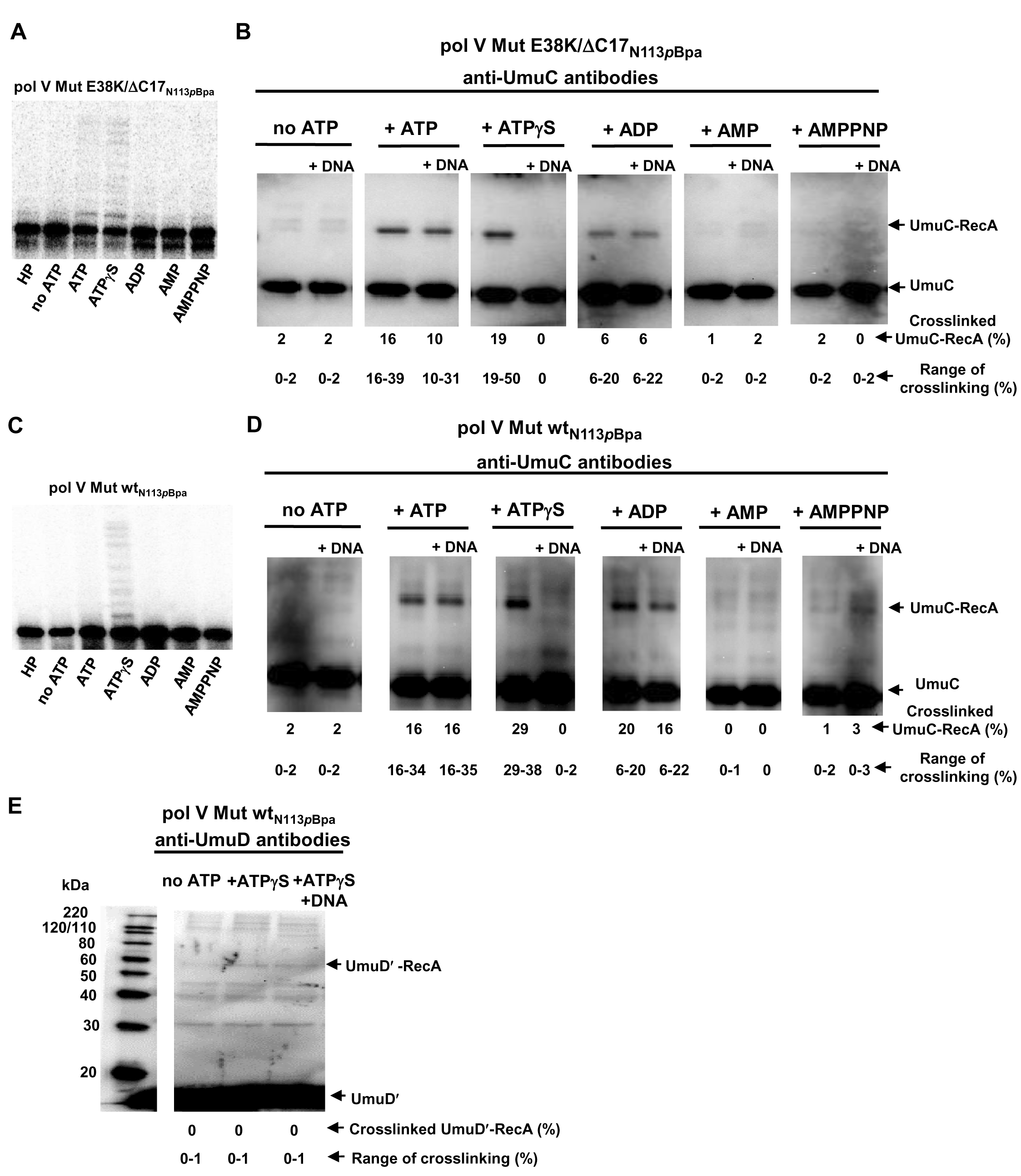

Supplement: S8 Fig — (A) Pol V Mut E38K/ΔC17N113pBpa activity with ATP, ATPγS, ADP, AMP, and AMPPNP. (B) UmuC-RecA crosslinking for pol V Mut E38K/ΔC17N113pBpa with ATP, ATPγS, ADP, AMP, and AMPPNP. RecA E38K/ΔC17N113pBpa forms crosslinks to UmuC in the presence of ATP, ATPγS, and ADP, but not with either AMP or AMPPNP. (C) Pol V Mut wtN113pBpa activity with ATP, ATPγS, ADP, AMP, and AMPPNP. (D) UmuC-RecA crosslinking for pol V Mut wtN113pBpa with ATP, ATPγS, ADP, AMP, and AMPPNP. RecA wtN113pBpa forms crosslinks to UmuC in the presence of ATP, ATPγS, and ADP, but not with either AMP or AMPPNP. (E) Absence of crosslinking between RecA wtN113pBpa and UmuD' for pol V Mut wt. This result was previously reported in Gruber et. al 2015 [12] and repeated here. Each crosslinking experiment was repeated 4 times. (TIF) [file pgen.1007956.s008.tif]

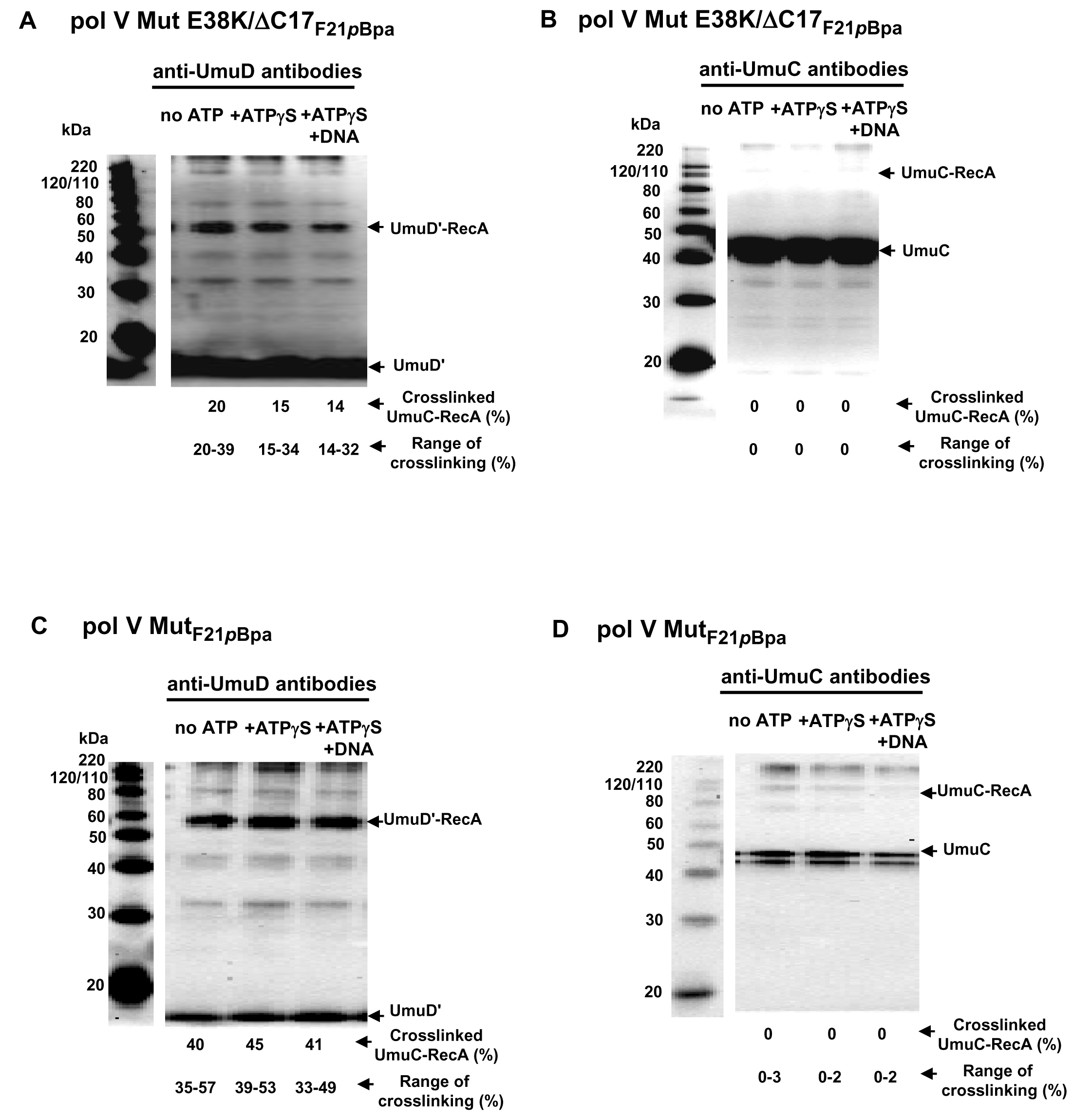

Supplement: S9 Fig — Pol V Mut was assembled with crosslinkable (A-B) RecA E38K/ΔC17F21pBpa and (C-D) RecA wtF21pBpa. RecA E38K/ΔC17F21pBpa (A) and RecA wtF21pBpa (D) crosslinks to UmuD' of pol V Mut. There is no crosslinking observed between either RecA E38K/ΔC17F21pBpa (B) or RecA wtF21pBpa (D) and UmuC. Each crosslinking experiment was repeated 3 times. (TIF) [file pgen.1007956.s009.tif]
